# Mass spectrometry-based affinity capture of peptide ligands from complex venom mixtures

**DOI:** 10.3389/fchem.2026.1971257

**Published:** 2026-10-27

**Authors:** Lou Freuville, Rudy Fourmy, Aude Violette, Alain Brans, Romulo Aráoz, Nicolas Gilles, Loïc Quinton

**Affiliations:** 1 Mass Spectrometry Laboratory, MolSys Research Unit, Department of Chemistry, University of Liège, Liège, Belgium; 2 Alphabiotoxine Laboratory srl, Montroeul-au-Bois, Belgium; 3 Centre for Protein Engineering, University of Liège, Liège, Belgium; 4 CEA, Paris-Saclay University, Gif-sur-Yvette, France

**Keywords:** affinity capture, animal venoms, ligand fishing, mass spectrometry, membrane receptors

## Abstract

Animal venoms constitute a rich source of bioactive peptides targeting membrane receptors, including G protein-coupled receptors (GPCRs) and ligand-gated ion channels. However, identifying receptor-binding ligands directly from complex venom mixtures remains analytically challenging because venoms are highly diverse and conventional screening approaches have limited throughput. Here, we present a membrane-based affinity-capture workflow coupled to mass spectrometry to recover known receptor ligands and prioritize additional venom toxins for validation. The methodology combines native membrane preparations, label-free affinity capture, MALDI-TOF mass spectrometry, and LC-MS/MS-based proteomic identification within a single analytical workflow. Its performance was demonstrated using two pharmacologically distinct membrane receptor families: the human vasopressin type 2 receptor, a G protein-coupled receptor, and the muscle-type nicotinic acetylcholine receptor, a ligand-gated ion channel. The workflow recovered known ligands spanning endogenous peptides and disulfide-rich venom toxins and documented AVP-dependent changes in toxin recovery. Direct comparison of bound fractions obtained with nAChR-rich *Torpedo* membranes and control CHO membranes further identified toxins preferentially retained by each membrane preparation. By combining receptor-guided affinity selection with direct molecular identification, this strategy provides a versatile analytical platform for investigating ligand–receptor interactions and prioritizing bioactive peptides from highly complex natural mixtures. Beyond venom research, the approach is readily adaptable to other membrane receptors and molecular libraries for ligand discovery. Proteomic data are available via ProteomeXchange with identifier PXD084762.

## 1 Introduction

Membrane receptors constitute one of the largest classes of therapeutic targets because they regulate numerous physiological processes through the recognition of endogenous and exogenous ligands. Among them, G protein-coupled receptors (GPCRs) represent the largest receptor family, with approximately 800 receptors encoded in the human genome, accounting for nearly 35% of all approved drug targets (Santos et al., 2017). Ligand-gated ion channels constitute another major receptor class involved in neuronal signaling, muscle contraction, and sensory transmission (Raines et al., 2022). The identification of ligands capable of selectively interacting with these receptors remains a central objective in pharmacology and drug discovery. However, discovering receptor-binding molecules directly from highly complex biological samples continues to represent a significant analytical challenge.

Animal venoms constitute an exceptional source of receptor-targeting ligands. More than 200,000 venomous animal species have been described, each potentially producing hundreds of bioactive peptides and proteins (Herzig et al., 2020). These molecules have evolved over millions of years to interact with membrane receptors with remarkable affinity and selectivity (Schendel et al., 2019). Their structural diversity and pharmacological specificity have made them invaluable tools for studying receptor function and have already led to several approved therapeutics for hypertension, chronic pain, diabetes, thrombosis, and coagulation disorders (Bordon et al., 2020; Freuville et al., 2024). Despite this exceptional molecular diversity, only a small proportion of venom peptides have been pharmacologically characterized, largely because identifying receptor-specific ligands within such complex mixtures remains experimentally demanding.

Mass spectrometry has become a powerful analytical platform for investigating ligand–receptor interactions owing to its label-free detection, high sensitivity, and ability to provide direct molecular identification (Hernández and Robinson, 2007; Wu et al., 2025). Several complementary MS-based approaches, including native MS, hydrogen-deuterium exchange MS, cross-linking MS, and affinity selection MS, have considerably expanded the possibilities for studying non-covalent protein–ligand interactions (Zhang et al., 2011; Herzog et al., 2012; Prudent et al., 2021). In parallel, receptor-based affinity methodologies, such as cellular membrane affinity chromatography, have demonstrated that membrane receptors can be exploited for ligand screening while preserving a biologically relevant membrane environment (Moaddel and Wainer, 2009). More recently, advances in venomics have enabled comprehensive characterization of venom composition through high-throughput combinations of chromatographic fractionation, intact mass profiling, shotgun proteomics, and automated data processing (Slagboom et al., 2023). However, these approaches primarily provide compositional information and generally do not directly identify which venom components interact with a specific molecular target.

Previous studies from our laboratory demonstrated the feasibility of membrane-based affinity capture monitored by mass spectrometry for identifying peptide ligands from venom fractions (Cologna et al., 2015). This strategy enabled direct observation of receptor–ligand interactions without radiolabeling or fluorescent tagging and subsequently led to the discovery of EIIB, a novel nicotinic acetylcholine receptor ligand from *Conus ermineus* venom (Echterbille et al., 2017). Together, these studies established membrane-based affinity capture for GPCR and nAChR ligands, including its use with complex peptide mixtures and venom fractions. Its application to larger disulfide-rich snake toxins and to crude snake venom analyzed by quantitative proteomics remained to be examined.

In the present study, we extended membrane-based affinity capture coupled to mass spectrometry to larger disulfide-rich snake toxins and examined their recovery from fractionated and crude venoms. The methodology combines native membrane preparations, label-free affinity capture, MALDI-TOF mass spectrometry, and LC-MS/MS-based shotgun proteomics within a single analytical workflow. Its analytical performance was evaluated using two pharmacologically distinct membrane receptor families, the human vasopressin type 2 receptor (hV2R), a G protein-coupled receptor, and the muscle-type nicotinic acetylcholine receptor (nAChR), a ligand-gated ion channel. The proposed workflow enables the selective recovery and molecular identification of ligands ranging from endogenous peptides to approximately 7.5 kDa disulfide-rich venom toxins from both fractionated and crude venoms, providing a versatile analytical platform for receptor-oriented ligand discovery.

## 2 Materials and methods

### 2.1 Receptors of interest

Membrane preparation expressing the hV2R were purchased from PerkinElmer (Waltham, USA) (B_max_ 11.5 pmol/mg membrane protein, product number: 6110541400UA), aliquoted, and stored at −80 °C. The receptor was overexpressed in the Chinese hamster ovary (CHO-K1) cell line. CHO membranes without any overexpressed receptor were also purchased from PerkinElmer (product number: RBCCHOM010MG), as a negative control. The nicotinic acetylcholine receptor (nAChR), muscle subtype (α1)_2_β1γδ, is naturally produced in *Torpedo marmorata* electrocyte membranes. Previous studies have estimated that nAChRs account for approximately 20%–40% of the total membrane protein content in these preparations (Changeux, 2012). Membrane preparations were obtained from our collaborators, Dr. Romulo Aráoz and Dr. Nicolas Gilles, from the CEA (Paris-Saclay, France).

*Torpedo marmorata* electrocyte membranes rich in nAChRs were purified as previously described (Aráoz et al., 2020). Briefly, the electrocyte membrane was dissected from a sacrificed *T. marmorata* adult fish according to the guidelines of the Ethical Committee for C2EA-59 of Paris Center-South 34175-20211130122692. The whole purification process was performed in a cold room at 4 °C. The electric organ (500 g) was homogenized in 500 mL of *Torpedo*-membrane extraction buffer (TMEB) containing 50 mM Tris-HCl, 3 mM EDTA, 1 mM EGTA, and protease inhibitors (Complete Roche Diagnostics GmbH, Mannheim, Germany) (pH 7.5) with a Waring blender (Appareils Scientifiques O.S.I., Paris, France) at maximal speed for 1 min (3 times with 1 min intervals). The homogenate was centrifuged at 4,000 g for 10 min at 4 °C (Sorvall, Dupont Instruments, Newtown, U.S., GSA rotor). The supernatant was filtered through several layers of gauze. The pellet was re-homogenized in 250 mL TMEB and processed as previously described. The supernatants were pooled and centrifuged at 25,000 g for 50 min at 4 °C (Sorvall, rotor GSA). The resulting pellet was resuspended in 300 mL TMEB supplemented with 35% sucrose (w/w) and homogenized using a Potter-Elvehjem (glass/Teflon). The electrocyte membranes were purified by sucrose density gradient ultracentrifugation by layering 16 mL of the membrane suspension over 10 mL of TMEB supplemented with 43% sucrose (w/w). The whole was ultracentrifuged at 106,000 g for 3 h at 4 °C (Beckman, Palo Alto, CA, U.S., 50Ti rotor). *Torpedo*-electrocyte membranes were collected from the interphase between the 35% and 43% sucrose-TMEB layers. The electrocyte membranes were precipitated by ultracentrifugation at 106,000 g for 1 h at 4 °C (Beckman, 50Ti). The electrocyte membranes were resuspended in 100 mL of 5 mM glycine using the glass/Teflon homogenizer and were ultracentrifuged as described. This washing step was repeated once more. The resulting pellet was resuspended in 40 mL of 5 mM glycine. The protein concentration was determined by the Bradford method in the presence of 1 M urea. One-milliliter aliquots of *Torpedo*-electrocyte membranes containing 3.5 mg/mL total protein were conserved at −80 °C until use.

### 2.2 Affinity capture of toxin ligands

The experimental workflow is outlined in Figure 1 (inspired by (Echterbille et al., 2017)). In all experiments, low-binding microcentrifuge tubes (Sigma-Aldrich®, Steinheim, Germany) were initially coated with 0.1% (w/v) bovine serum albumin (BSA) to minimize nonspecific peptide and protein adsorption onto the plastic surface. Subsequently, receptor-containing membrane preparations and a mixture of peptides/toxins to be tested were added to a binding solution composed of 50 mM ammonium acetate (pH 7.4) supplemented with 5 mM magnesium chloride. The final volume for each assay was standardized to 20 µL. Samples were incubated at 27 °C for 2 h under reduced agitation (400 rpm) to promote ligand-receptor interactions, while unbound peptides remained in solution. Following incubation, samples were centrifuged at 15,600 g for 3 min at 4 °C. The resulting supernatant, containing the unbound peptides, was carefully collected and designated as the “Unbound Fraction” (UF) throughout the study (Figure 1A). The pellet, containing membrane receptor-ligand complexes, was washed with a salt-free 50 mM ammonium acetate solution, pH 7.4 (Figure 1B). After resuspension and rinsing, the sample was centrifuged again under identical conditions, and the washing solution was discarded. For the ligand elution, the pellet was resuspended in a 5% (v/v) trifluoroacetic acid (TFA) solution, adjusted to pH 2. This acidic treatment disrupted receptor structure, leading to the release of the bound ligands. A final centrifugation step was performed under the same parameters, and the supernatant, corresponding to the “Bound Fraction” (BF), was collected for further analyses. Both unbound and bound fractions were desalted using ZipTip® C18 pipette tips (8 μg, Pierce, Rockford, IL, USA) to remove residual salts. Peptide elutions were performed with 5 µL of a 50/50 (v/v) acetonitrile:0.1% formic acid (FA) solution. The purified samples were analyzed by matrix-assisted laser desorption/ionization time-of-flight mass spectrometry (MALDI-TOF-MS), and, for some complex samples, by liquid chromatography-tandem mass spectrometry (LC-MS/MS) (see below).

**FIGURE 1:**
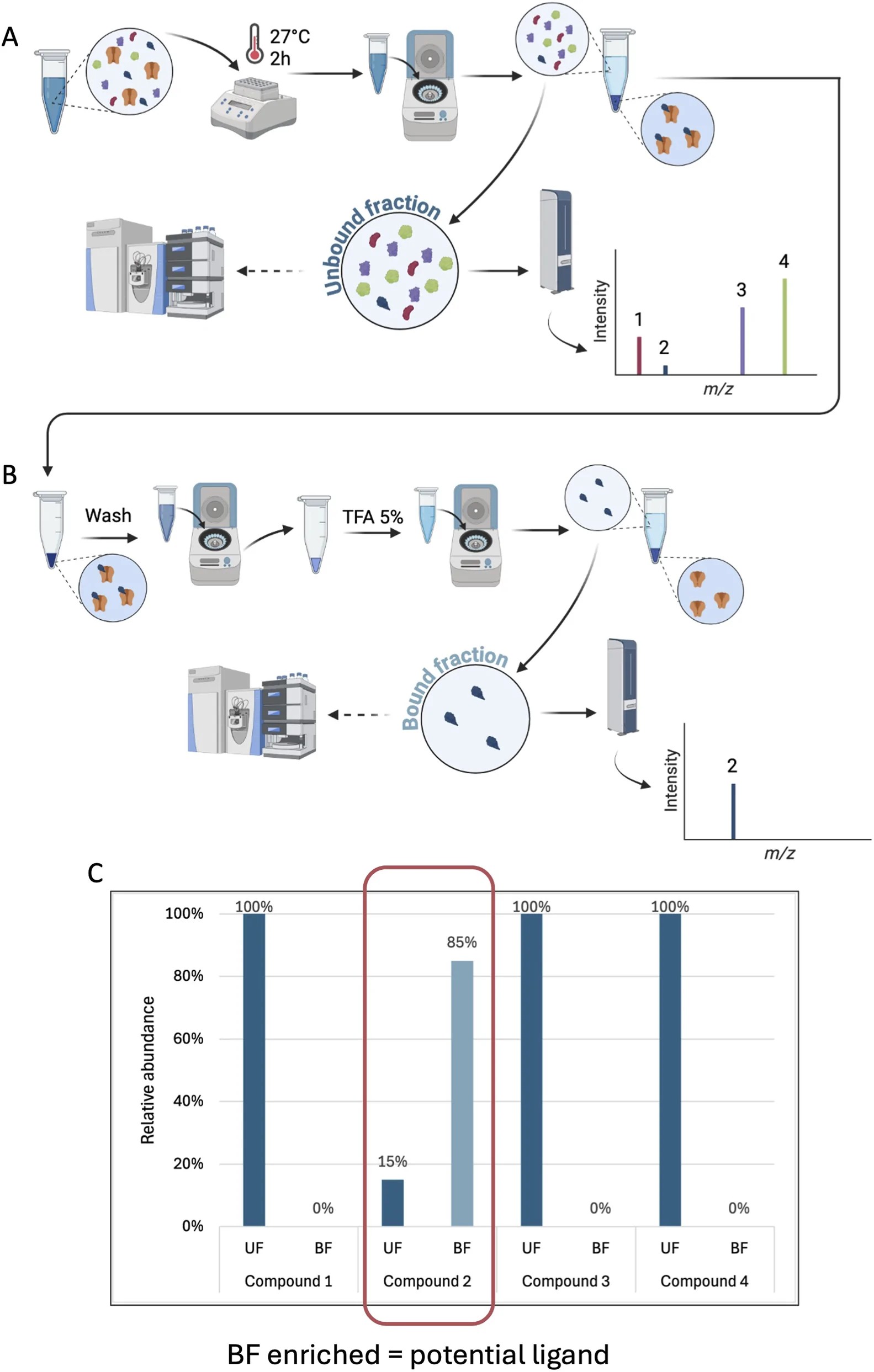
Methodology for affinity capture of peptide toxins. **(A)** Peptides and membranes containing the receptor of interest are mixed and incubated at 27 °C for 2 h. Centrifugation was performed to isolate the peptides that did not bind to the receptor in the supernatant. After centrifugation, unbound peptides were recovered in the supernatant, which was collected, desalted, and analyzed by MALDI-TOF MS. **(B)** The pellet was washed and then eluted with an acid solution to disrupt the interaction between the bound peptides and the receptor. Centrifugation was performed to obtain the bound peptides in the supernatant. The supernatant was collected, desalted, and analyzed by MALDI-TOF-MS. More complex samples were also analyzed by LC-MS/MS. Created with BioRender.com (2026). **(C)** Histogram representation of relative peptide abundances in UF and BF. Enrichment in BF highlights compounds retained under the assay conditions for further investigation, while UF-enriched compounds showed little or no detectable retention under the tested conditions.

To describe the distribution of a detected MALDI-TOF signal between the separately collected unbound and bound fractions, relative signal intensities were calculated as follows:

$$Proportion\;Unbound\;fraction=100*\left(\frac{I\left(UF\right)}{I\left(UF\right)+I\left(BF\right)}\right)$$ (1)

$$Proportion\;Bound\;fraction=100*\left(\frac{I\left(BF\right)}{I\left(UF\right)+I\left(BF\right)}\right)$$ (2)

Because UF and BF were separately recovered, desalted, and spotted without an internal recovery standard, these normalized intensities were used to describe signal partitioning rather than the proportion of toxin molecules bound.

### 2.3 Control experiments

#### 2.3.1 Blank experiments

To evaluate the signals from the cellular matrix or assay components, control experiments were performed using the same binding protocol in the absence of potential ligands. These conditions allowed us to assess spectral baseline responses and isolate the specific contribution of cell membranes in the context of such binding experiments (incubation in a buffer, centrifugation, acidic treatment). In the mass spectrum of the blank, different compounds are detected in the bound fraction for the hV2R-overexpressing cell membranes (Supplementary Figures S1A and B) and for the *Torpedo* nAChR-containing cell membranes (Supplementary Figure S1C). These compounds, marked with an asterisk in subsequent figures, are not specific ligands but result from molecules released due to the acid shock introduced during the elution step. This control highlights that certain molecules can be nonspecifically released under acidic conditions, underscoring the importance of distinguishing them from true receptor-ligand interactions in the analysis.

#### 2.3.2 Positive control experiments

To ensure the reliability, specificity, and functional relevance of our binding assay, positive control experiments were systematically performed for each receptor studied. In these positive controls, a known high-affinity ligand was spiked into a complex peptide mixture (trypsin digest of bovine serum albumin protein, in the present study). The full protocol was then applied to assess whether the method could effectively isolate the known ligand from the complex peptide mixture based on its affinity for the target receptor. As discussed in the results section, successful retrieval of the spiked ligand confirmed that the system was performing as expected, detecting specific peptide-receptor interactions even in complex mixtures. The AVP positive control was performed alongside the hV2R capture experiments to verify ligand recovery with the membrane preparation used. The α-conotoxin GI positive control was performed repeatedly during the nAChR experiments. Representative control spectra are shown in Supplementary Figures S4 and S6.

For hV2Rs, the positive control experiment was performed using arginine vasopressin (AVP), the natural endogenous ligand of hV2Rs (K_D_ = 0.18 nM), as the positive binder. In this setup, 3 pmol of AVP was incubated with 200 fmol of hV2Rs, corresponding to 2.5 µL of membrane preparations. For nAChRs, 3 pmol of synthetic α-conotoxin GI (IC_50_ = 20 nM for nAChRs) (Ning et al., 2018) was incubated with 2.5 µL of *Torpedo* electrocyte membrane stock (3.5 mg/mL total protein), corresponding to 8.75 µg of membrane protein. In each experiment, 3 pmol of digested bovine serum albumin (BSA) (representing approximately 100 different detectable peptides) was added to the preparations to mimic a complex biological environment. The same amount of membrane preparation was used in the other affinity-capture assays. The receptor and ligand characteristics are summarized in Table 1.

**TABLE 1:** Receptors used for binding assays.

Studied receptors	Receptor environment	Known ligand (positive control)	Ligand sequence	Ligand molecular mass	Disulfide bridge	Affinity	References	Background
hV2R	Overexpressed CHO cell membranes	Arg-vasopressin (AVP)	CYFQNCPRG-NH_2_	1084.23	Cys1-Cys6	K_D_ = 0.18 nM	Postina et al. (1996)	Digested BSA
Muscle-type nAChRs (α1)_2_β1γδ	*Torpedo marmorata* electric organ preparation	α-conotoxin GI	ECCNPACGRHYSC-NH_2_	1437.6	Cys2-Cys7 Cys3-Cys13	IC_50_ = 20 nM	Ning et al. (2018)	Digested BSA

#### 2.3.3 Negative control experiments

Control affinity-capture experiments were performed with CHO membranes lacking overexpression of the receptor of interest. These followed the same experimental procedure as the main assays but were conducted on CHO cell membranes that did not express any receptor of interest. For the hV2R experiments, this condition provided a CHO membrane background without hV2R overexpression. For the nAChR experiments, CHO membranes were used to identify toxins that could also be retained by an unrelated membrane preparation. Because CHO and *Torpedo* electrocyte membranes differ in cellular origin, lipid composition, and endogenous proteins, this condition was treated as an exploratory counter-screen rather than a matched receptor-negative control for *Torpedo* membranes.

### 2.4 Fishing high-mass peptide ligands for hV2Rs: application to mambaquaretin-1

To demonstrate our methodology’s capability to capture high-mass peptide ligands, mambaquaretin-1 was selected as a model compound. MQ-1 is a peptide isolated from *D. angusticeps* venom and is known for its high affinity toward hV2R (K_i_ = 2.81 nM) (Ciolek et al., 2017). An MQ-1-containing fraction (Alphabiotoxine laboratory, Montroeul-au-Bois, Belgium) was obtained from crude *Dendroaspis angusticeps* venom by reverse-phase liquid chromatography. Briefly, the venom was first subjected to a C18 solid-phase extraction using a SepPak C18 cartridge (Waters, Milford, MA, USA) to remove non-retained components. The retained material was eluted with 70% acetonitrile containing 0.1% TFA, freeze-dried, and reconstituted before fractionation. Reverse-phase fractionation was then performed on a Vydac 218TP C18 column (5 µm particle size) at a flow rate of 0.8 mL/min. Following sample injection, peptides were separated using an acetonitrile gradient in 0.1% TFA at 0.8 mL/min. The acetonitrile concentration increased from 0% to 72% over 80 min, then from 72% to 90% over 10 min, followed by a 10-min hold at 90%. The column was subsequently re-equilibrated with five column volumes of 0.1% TFA at 1.5 mL/min. Fractions of 1 mL were collected throughout the separation, freeze-dried, and stored until use. The resulting chromatogram is available in Supplementary Figure S2. The fraction containing MQ-1 (fraction D1), previously identified by mass spectrometry, was used for ligand-fishing experiments. For affinity-capture assays, 3 µL of the MQ-1-containing fraction was added to the receptor preparation. This same fixed amount of the MQ-1-containing fraction was used in competition assays, in which AVP (the endogenous hV2R ligand) was added at increasing concentrations (1, 5, 10, 50, 75, and 100 pmol) to examine AVP-dependent changes in toxin recovery. Following incubation, samples were processed as previously described, and the bound and unbound fractions were analyzed using MALDI-TOF MS. Fraction D1 was tested in a capture assay without added AVP and in a separate AVP concentration-series assay, with one capture at each concentration.

### 2.5 Toxin ligand-fishing for nAChRs

As for hV2Rs, the workflow was expanded to explore natural ligands of nAChRs found in complex venom mixtures. For this purpose and to compare the efficacy of ligand capture in whole venom versus fractions, *N. kaouthia* crude venom was used, as well as venom fractions (Alphabiotoxine laboratory, Montroeul-au-Bois, Belgium). The venom was fractionated by HPLC. 1 mg of *Naja kaouthia* venom was first reconstituted in H_2_O/0.1% TFA and separated using a C18 Supelco Discovery BIO wide pore 3 µm column (150 × 2.1 mm) (Sigma-Aldrich®, Steinheim, Germany). Before fractionation, the sample was centrifuged at 8,000 rpm for 20 min. The supernatant was transferred to a clean Eppendorf tube. Fractionation was performed on an Alliance HPLC system (Waters Corporation, Milford, MA, USA) at a flow rate of 0.3 mL/min. UV absorbance was monitored at 214 and 280 nm. The mobile phases consisted of water containing 0.1% TFA (solvent A) and acetonitrile containing 0.1% TFA (solvent B). The column was equilibrated at 2% solvent B. After sample injection, the solvent composition was maintained at 2% B for 5 min, followed by a linear increase from 2% to 65% B between 5 and 70 min. The column was then washed at 90% B for 10 min and either stored or re-equilibrated under the initial conditions before the next run. Fractions of 0.3 mL were collected in 96-well plates using an automatic fraction collector, then dried using a SpeedVac and stored at −20 °C. The resulting chromatogram is available in Supplementary Figure S3. For this ligand-fishing, 1.5 µg of crude venom, or 3 µL of different HPLC fractions, was added to the receptor preparation.

### 2.6 MALDI-TOF/TOF mass spectrometry

#### 2.6.1 Sample preparation

Two different MALDI sample preparation methods were employed. In both protocols, matrix solutions were vortexed and centrifuged before use to ensure homogeneity. For most analyses, including nAChR ligand-fishing experiments and hV2R assays performed with arginine vasopressin (AVP), samples were prepared using an ultra-thin layer of α-cyano-4-hydroxycinnamic acid (CHCA), applied to enhance resolution and ensure uniform spatial distribution (Fenyo et al., 2007). For that purpose, a CHCA solution saturated in acetone was first spotted onto a MALDI target plate (MTP 384 ground steel target, Bruker, Billerica, MA, USA) to form the base layer. A second matrix layer was then applied, consisting of CHCA at 20 mg/mL in a 70:30 (v/v) acetonitrile-acidified water mixture (5% formic acid). Once this layer dried, 1 μL of the sample was deposited onto the prepared spot, followed by 1 μL of the matrix solution to promote efficient co-crystallization.

For experiments involving MQ-1-containing fractions, samples were prepared using the dried-droplet method with 2,5-dihydroxybenzoic acid (DHB) as the matrix. The matrix solution was prepared by dissolving DHB at 20 mg/mL in a 50:50 (v/v) mixture of acetonitrile and acidified water (0.1% formic acid), followed by mixing and centrifugation to ensure homogeneity. For sample preparation, 1 μL of the sample was deposited onto the MALDI target plate and immediately overlaid with 1 μL of the matrix solution. The mixture was then allowed to dry at ambient temperature, enabling co-crystallization of analytes and matrix components.

#### 2.6.2 Data acquisition and analysis

All MALDI-TOF mass spectrometry acquisitions were performed using a RapifleX® instrument (Bruker, Billerica, MA, USA). MS spectra were acquired in either reflector or linear positive mode, depending on the specific experimental requirements. For each sample, five acquisitions were performed, consisting of 1,000 laser shots per spot in reflector mode and 5,000 laser shots per spot in linear mode. The laser intensity was maintained at a constant energy and fluence within each experiment to ensure consistency across samples. Spectral data were analyzed using FlexAnalysis 3.4 (Bruker Daltonics). Instrument calibration was carried out using either the Peptide Calibration Standard (Bruker Daltonics), comprising seven peptides spanning a 700–3,500 Da mass range, or the Protein Calibration Standard I (Bruker Daltonics), containing four proteins in the 4,000–20,000 Da range, depending on the experimental context. For calibration, 0.5 μL of the standard solution was mixed with 1 μL of matrix solution and spotted onto the MALDI plate.

### 2.7 Shotgun proteomics analysis

#### 2.7.1 Sample preparation

Samples of interest were first dried using a speed vacuum system and resuspended in 20 µL of 50 mM ammonium bicarbonate (NH_4_HCO_3_) buffer at pH 7.8. Disulfide bond reduction was performed by adding 2 µL of dithiothreitol (DTT) to get a final concentration of 10 mM, followed by incubation at 56 °C for 1 h with agitation (400 rpm). Subsequently, cysteine residues were alkylated by adding 3 µL of iodoacetamide (IAA) to reach a final concentration of 20 mM and incubating for 45 min at room temperature in the dark. To quench the alkylation reaction and avoid any side reactions, 2 µL of 11 mM DTT was added and incubated for 10 min at room temperature, also in the dark. Proteolytic digestion was performed in two consecutive steps using trypsin. The first digestion used a trypsin-to-protein ratio of 1:50 and was carried out overnight at 37 °C under agitation (400 rpm). The second digestion, performed the following day, used a 1:100 ratio and included the addition of acetonitrile to a final concentration of 80% (v/v) to promote protein unfolding and enhance enzymatic efficiency. This step was incubated for 3 h at 37 °C. Proteolysis was stopped by acidification with 10% (v/v) trifluoroacetic acid (TFA), and samples were dried using a speed vacuum.

Before mass spectrometry analysis, peptides were resuspended in 20 µL of 0.1% TFA and desalted using ZipTip C18 pipette tips. Elution was performed with 10 µL of 50:50 (v/v) acetonitrile:0.1% TFA. The eluates were dried again under vacuum and finally reconstituted in 0.1% TFA for analysis.

For the comparisons of crude *N. kaouthia* venom, three affinity-capture experiments were performed in parallel for each membrane condition using the same membrane preparation and venom stock (n = 3 experimental replicates). The resulting BF and UF samples were digested separately, yielding three paired BF–UF samples per condition. Three aliquots of the same crude-venom stock were digested separately as VB sample-preparation replicates (n = 3 technical replicates).

#### 2.7.2 Data acquisition

Generated peptides were separated and characterized using a liquid chromatography-tandem mass spectrometry (LC-MS/MS) system consisting of an Acquity M-Class UPLC (Waters Corporation, Milford, MA, USA) coupled to a Q-Exactive Plus mass spectrometer (Thermo Scientific, Waltham, MA, USA) in nano-electrospray positive ion mode. The chromatographic setup included a Symmetry C18 trap column (5 μm, 180 μm × 20 mm) and an analytical column (HSS T3 C18, 1.8 μm, 75 μm × 250 mm) from Waters (Milford, MA, USA). Samples were initially loaded onto the trap column at a flow rate of 20 μL/min in 98% solvent A (0.1% formic acid in water) for 3 min. Chromatographic separation was then carried out on the analytical column at 500 nL/min using a gradient elution starting at 98% solvent A, followed by a decrease to 93% over 5 min, 70% at 60 min, and 60% at 70 min, with a final hold at 10% A for 5 min before re-equilibration. Solvent B consisted of 0.1% formic acid in acetonitrile, and the total runtime was 100 min.

Mass spectrometric data were acquired using a TopN data-dependent acquisition method with N set to 12. The 12 most intense precursor ions (excluding singly charged and unassigned species) were selected for fragmentation and subsequent MS/MS acquisition. The parameters for MS spectrum acquisition are as follows: mass range: 400–1600 *m/z*; resolution: 70,000; AGC target: 3 × 10^6^, or maximum injection time: 50 m. The parameters for MS/MS spectrum acquisition are: Isolation Window of 2.0 m/z; normalized collision energy (NCE) of 28; resolution of 17,500; AGC target of 1 × 10^5^ or maximum injection time of 50 m. The main parameters for the Q Exactive tune were a spray voltage of 2.3 kV, a capillary temperature of 270 °C, and an S-Lens RF level of 50.0.

The mass spectrometry proteomics data have been deposited to the ProteomeXchange Consortium via the PRIDE (Perez-Riverol et al., 2025) partner repository with the dataset identifier PXD084762 and 10.6019/PXD084762.

#### 2.7.3 PEAKS X+ bioinformatics data analysis

Protein identification was performed using PEAKS X + Studio 10.5 software by searching MS/MS spectra against the “*D. angusticeps*” database (UniProt, 78 sequences, June 2026) for the experiment with *D. angusticeps* venom, and the “*N. kaouthia*” database (UniProt, 216 sequences, April 2026) for the experiment with *N. kaouthia* venom. Carbamidomethylation (C) was designated as a fixed modification, whereas oxidation of methionine (M) was set as a variable modification. A maximum allowance of three missed cleavages was specified per protein. Mass error tolerances were established at 5 ppm for precursor ions (MS) and 0.015 Da for fragment ions (MS/MS).

To ensure data reliability, a false discovery rate (FDR) of 0.1% was applied. Proteins were filtered using PEAKS search algorithms requiring at least one unique peptide and significant peptides per identified protein to eliminate false-positive identifications. Confident database matches were defined by a score threshold of −10LgP >20, while proteins were considered relevant if they achieved a score of 20 or higher. The score was derived by taking −10 times the logarithm (base 10) of the p-value. Unique peptides, which are exclusive to specific protein groups and absent from others, were crucial for accurate identification. To ensure robustness, each protein group required at least one unique peptide. Individual toxin accessions were therefore reported only when the peptide evidence supported their discrimination from other database entries. When the detected peptides were compatible with several closely related toxins, the corresponding accessions were retained as a protein group. Top-ranked proteins were prioritized by the number of unique peptides supporting their identification in each group.

#### 2.7.4 MaxQuant and perseus-based bioinformatics data analysis

Raw LC–MS/MS datasets generated on the Q-Exactive Plus mass spectrometer were analyzed using MaxQuant (v2.7.5.0) coupled to the integrated Andromeda search engine. Spectra were searched against the “*N. kaouthia*” protein database retrieved from UniProt (216 sequences, accessed in April 2026). Database searching was performed using specific digestion with Trypsin/P, allowing up to two missed cleavages. Carbamidomethylation of cysteine residues (C) was defined as a fixed modification, while methionine oxidation (M) was included as a variable modification. Default mass tolerance parameters implemented in MaxQuant were applied for precursor and fragment ion matching. The minimum peptide length was set to six amino acids. Label-free quantification (LFQ) was enabled with a minimum ratio count of 1. Protein and peptide identifications were filtered according to the default false discovery rate criteria implemented in MaxQuant.

Proteomic data processing and statistical analyses were performed using Perseus (v2.1.6.0). Entries annotated as “only identified by site”, “reverse hits”, or “potential contaminants” were excluded. LFQ intensities were log_2_-transformed. For each proteomic comparison, protein groups were retained when at least two of three LFQ values were measured in at least one of the two groups before imputation. Remaining missing values were imputed from a normal distribution using a width of 0.3 and a downshift of 1.8. Volcano plot analyses were performed on log_2_ LFQ intensities. Differential abundance was assessed using Perseus two-sample tests comparing each bound fraction with crude venom and directly comparing *Torpedo* and CHO-BF. Statistical significance was determined using a permutation-based false discovery rate (FDR) of 1% and S0 = 0.5. Principal component analysis (PCA) was used to examine the distribution of proteomic samples. Quantitative analyses were performed at the protein-group level. Semicolon-separated accessions indicate closely related proteins that could not be resolved from the identified peptides.

## 3 Results

### 3.1 Detection and identification of toxin ligands for hV2R

Before investigating venom-derived ligands, the affinity-capture workflow was validated using the well-characterized hV2R–AVP ligand-receptor pair. These validation experiments confirmed the ability of the method to selectively recover a known peptide ligand from a complex mixture and are presented in Supplementary Material 1, Supplementary Figure S4.

#### 3.1.1 Affinity capture of MQ-1

Having established the validity of the affinity-capture workflow, the method was next applied to identify natural venom-derived ligands targeting hV2R. To our knowledge, such an affinity-based capture approach monitored by mass spectrometry has never been used to isolate medium-sized toxin peptides (M > 5 kDa), particularly in the context of GPCR recognition. This expands the application of receptor-based ligand fishing to the discovery of bioactive high-mass venom components. To first evaluate the feasibility of this approach, a ligand-fishing experiment was initiated using MQ-1, a known hV2R-targeting venom peptide. MQ-1 is indeed a 57-amino acid peptide isolated from the venom of the green mamba (*D. angusticeps*). It is the first natural peptide identified to selectively bind and antagonize the hV2R, making it a valuable pharmacological tool and a model ligand for GPCR-targeted toxin discovery (Droctové et al., 2022).

To assess MQ-1 capture, the venom fraction of *D*. *angusticeps*, containing MQ-1, was incubated with hV2R membrane preparations (venom fraction D1, see Supplementary Figure S2). As shown in the reference spectrum (Figure 2A, top), this fraction contains at least ten toxins, exhibiting masses from 6 to 9 kDa. Both the unbound (Figure 2A, middle) and bound **(** Figure 2A, bottom) fractions were analyzed by MALDI-TOF mass spectrometry in linear mode to enhance the spectrometer sensitivity. By comparing the MS spectrum of the reference with the unbound one, a substantial decrease in the intensity of the MQ-1 signal is revealed, relative to the other toxins. In contrast, a clear enrichment of the MQ-1 signal is observed in the bound fraction, demonstrating that mambaquaretin-1 is specifically captured by hV2R membrane preparations from a complex venom fraction, thereby confirming the selectivity and efficiency of the affinity-based ligand-fishing approach even in the presence of multiple structurally related toxins.

**FIGURE 2:**
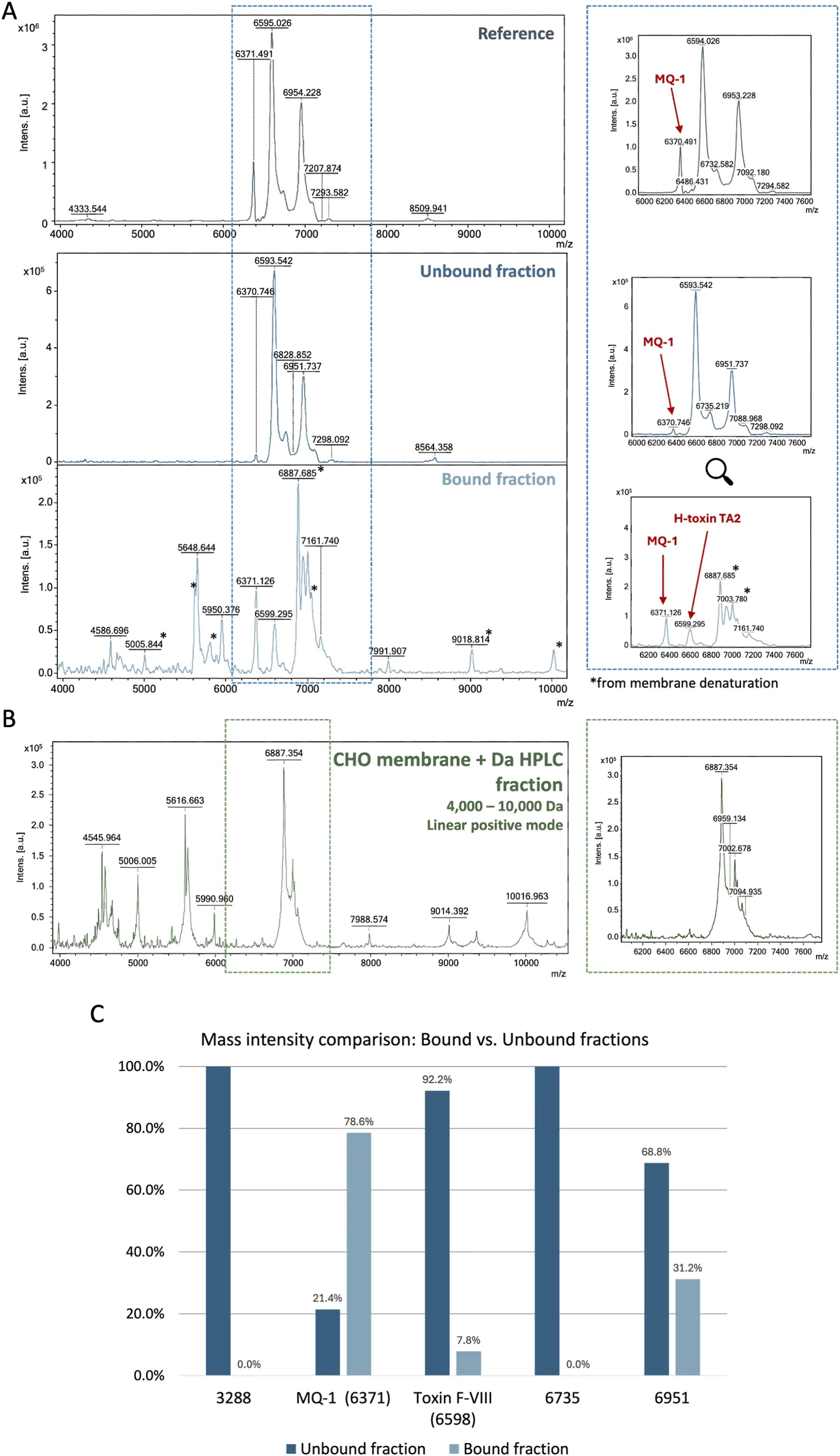
Affinity capture of mambaquaretin-1 on hV2R from a fraction of *Dendroaspis angusticeps* venom. **(A)** MALDI-TOF mass spectra comparing (i) the reference venom fraction (no receptor) at the working concentration, (ii) the UF, and (iii) the BF. In the UF, the MQ-1 peak is still detectable but shows a marked reduction in intensity compared to the reference, while the H-toxin TA2 peak remains intense. In the BF, MQ-1 enrichment is observed, with a decrease in the H-toxin TA2 signal. A zoomed-in view of the relevant m/z region is included to highlight these variations. Peaks marked with an asterisk (*) correspond to signals originating from membrane denaturation, as determined from the blank control (Supplementary Figure S1B). **(B)** MALDI-TOF mass spectra of the negative control performed with CHO membranes not expressing the receptor. **(C)** The bar graph shows the relative signal intensities of MQ-1 and H-toxin TA2 in the unbound (dark blue) and bound (light blue) fractions. Bars represent the relative intensity normalized to the sum of the two fractions, highlighting the selective capture of MQ-1 by hV2R.

The analysis of the MALDI-TOF spectra also revealed an additional, weaker signal in the bound fraction that was not initially expected based on the known pharmacology of the fraction. Subsequent proteomic identification associated this signal with the H-toxin TA2 (P01404). In contrast to MQ-1, the H-toxin TA2 signal remained predominantly detected in the unbound fraction, while only a weaker signal was observed in the bound fraction.

In the absence of hV2R expression (Figure 2B), neither MQ-1 nor H-toxin TA2 was detected in the bound fraction, showing that their retention differed between hV2R-expressing and control membrane preparations. Figure 2C shows the relative signal intensities of MQ-1 and H-toxin TA2 in both unbound and bound fractions. MQ-1 showed a preferential capture by the receptor, while H-toxin TA2 remained mostly unbound, confirming the selective enrichment of MQ-1. The ion detected at m/z 6951.7 was recovered in both the unbound and bound fractions. Unlike MQ-1, which was enriched in the bound fraction, and H-toxin TA2, which was predominantly detected in the unbound fraction, this signal showed a more balanced distribution between the two populations. As this peptide could not be confidently identified, its potential interaction with hV2R was not investigated further. Encouraged by the successful selective enrichment of MQ-1 from the *D. angusticeps* venom fraction, the same affinity-based capture strategy was applied to evaluate the direct fishing of ligands from the crude venom itself. Unfortunately, no MQ-1 was detected in the bound fraction (*data not shown*). This absence of enrichment suggests that prefractionation, even rough, is advised for better capture efficiency.

#### 3.1.2 Competition experiments between MQ-1 and AVP

To examine how AVP affects toxin recovery, a fixed volume of the RP-HPLC fraction containing MQ-1 was incubated with hV2R-expressing membranes in the presence of increasing amounts of AVP. As AVP increased, the MQ-1 signal decreased in the BF and increased in the UF (Figure 3A). This AVP-dependent redistribution is consistent with competitive displacement under the assay conditions but does not, by itself, identify the toxin-binding site. H-toxin TA2, detected in the same fraction, showed a much smaller BF signal in the absence of added AVP: less than 20% of its combined BF and UF MALDI signal was detected in the BF (Figure 3B). Its BF signal also decreased as AVP increased. Because these values describe normalized MALDI signals rather than the proportion of toxin molecules bound, they cannot establish the binding affinity or binding site of H-toxin TA2. Although the competition profile showed an apparent transition at AVP concentrations in the micromolar range (approximately 10^–6^ M), this observation should not be directly compared with the radioligand displacement assay reported by Ciolek et al., in which increasing concentrations of purified MQ-1 displaced a fixed concentration of radiolabeled AVP (^3^H-AVP), yielding a K_i_ of 2.81 nM (Ciolek et al., 2017). In contrast, the present experiment monitored the recovery of MQ-1 in the bound fraction by MALDI-TOF MS while competing with increasing concentrations of AVP. Furthermore, the experiments were performed using an RP-HPLC venom fraction rather than purified MQ-1, and the actual concentration of MQ-1 within this fraction is unknown. Additional co-eluting venom components may also affect toxin recovery and detection. Consequently, the observed competition profile reflects the collective behavior of the venom fraction rather than the intrinsic binding affinity of purified MQ-1. Finally, this experiment was performed as an exploratory proof-of-concept without independent affinity-capture replicates and with a limited number of concentration points. The corresponding nonlinear regression obtained with GraphPad Prism is therefore provided only as Supplementary Material (Supplementary Figure S5) and should be considered as a qualitative illustration of AVP-dependent signal redistribution rather than a quantitative determination of binding parameters. Quantitative estimation of competition parameters would require purified MQ-1, additional concentrations around the apparent transition, and independent repetitions of the competition assay.

**FIGURE 3:**
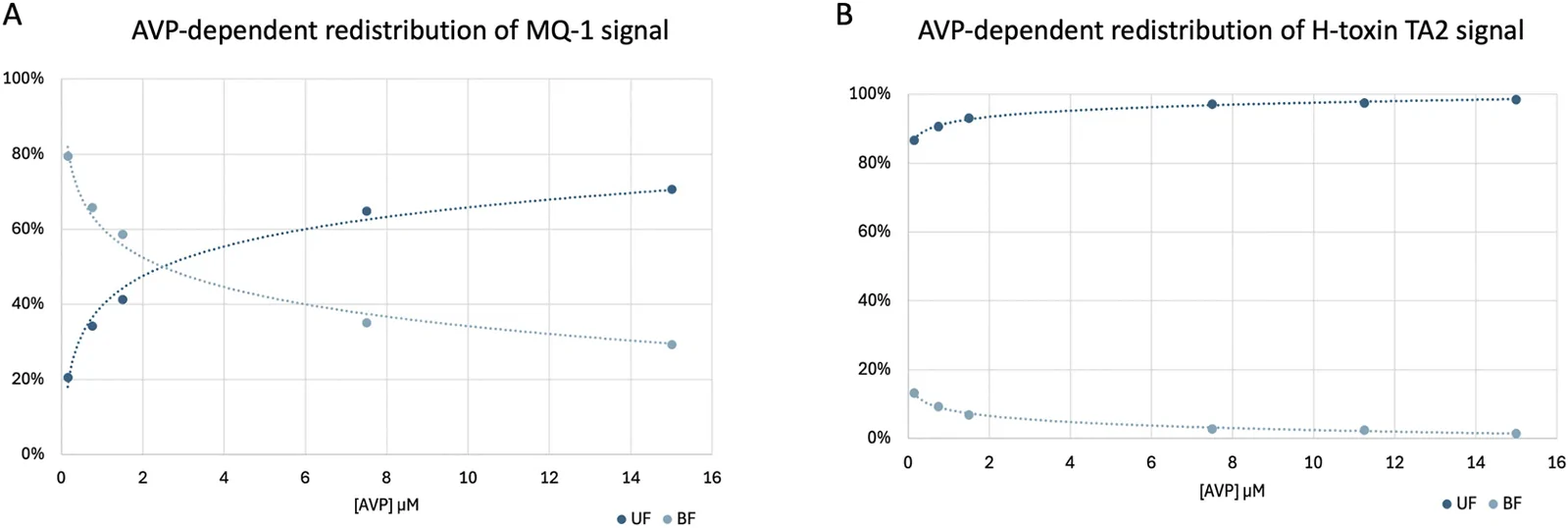
AVP-dependent redistribution of MQ-1 and H-toxin TA2 signals in affinity-capture experiments with hV2R-expressing membranes. A fixed volume of the RP-HPLC fraction containing MQ-1 was incubated with increasing amounts of AVP. Relative MALDI-TOF signal intensities in the UF and BF, normalized to their sum for each condition, are shown for **(A)** MQ-1 and **(B)** H-toxin TA2 as a function of AVP concentration. The BF signals of both toxins decreased upon AVP addition. Dotted lines are qualitative fits to the experimental data.

### 3.2 Detection and identification of toxin ligands for nicotinic acetylcholine receptors

The affinity-capture workflow was validated on nAChR-enriched *T. marmorata* membranes using α-conotoxin GI, a well-characterized nAChR ligand. These validation experiments confirmed the selective recovery of a known receptor-binding peptide and are presented in Supplementary Material 2; Supplementary Figure S6.

#### 3.2.1 Affinity capture of neurotoxins from *Naja kaouthia* venom

Having successfully validated the methodology using α-GI, the next objective was to evaluate the robustness and applicability of the approach with more complex and less defined biological samples. For this purpose, the venom of *N. kaouthia* (monocled cobra) was selected. This medically relevant elapid snake, widely distributed across South and Southeast Asia, is responsible for numerous envenomation cases in these regions. This lethality is mainly due to its venom composition, particularly rich in α-neurotoxins targeting nAChRs (Laustsen et al., 2015). This property makes it a biologically relevant and challenging matrix for evaluating the selectivity and sensitivity of our receptor-based affinity approach. This transition represents a critical step in assessing the method’s ability not only to confirm binding of high-mass ligands on nAChRs but also to selectively enrich and identify bioactive peptides directly from a complex biological mixture. Beyond *N. kaouthia*, homologous toxins have also been characterized in other *Naja* species, providing a broader framework for interpreting the identified components. The well-characterized toxin repertoire of *N. kaouthia* therefore makes this venom a suitable complex matrix for evaluating the workflow.

As a first step, discrete venom fractions were selected for screening rather than the crude venom itself. This stepwise approach reduces sample complexity and increases the likelihood of detecting specific interactions with the receptor. The venom was fractionated by RP-HPLC, yielding a total of 62 fractions based on their elution profiles (Supplementary Figure S3). Fractions corresponding to the most intense chromatographic peaks (F25, F31, and F42), together with consecutive fractions spanning the α-cobratoxin elution window (F31 and F35–F39), were selected for subsequent affinity-capture experiments. MALDI-TOF MS spectra were acquired in linear positive mode for each selected fraction following the fishing assay, and the signal intensities of detected masses were extracted from the unbound and bound fractions (Supplementary Figures S7–S9). For each detected mass, signal intensities were normalized to the total signal measured across both fractions. In parallel, shotgun proteomics experiments were performed on the bound fractions to identify the observed m/z signals. The experimental masses detected by MALDI-TOF MS, together with their corresponding toxin identifications when confidently assigned by proteomic analysis, are summarized in Table 2.

**TABLE 2:** Experimental MALDI-TOF MS masses detected following affinity capture, together with corresponding toxin identifications when available from shotgun proteomics.

Number	Experimental average mass	Accession number (UniProt)	Name	Species	Theoretical natural average mass (Da)	Cytotoxicity	Binding to nAChRs	References
1	5548.0	—	​	​	​	​	​	​
2	5563.9	—	​	​	​	​	​	​
3	5936.9	—	​	​	​	​	​	​
4	6627.5	—	​	​	​	​	​	​
5	6638.9	—	​	​	​	​	​	​
6	7128.8	—	​	​	​	​	​	​
7	13,225.3	—	​	​	​	​	​	​
8	13,404.9	—	​	​	​	​	​	​
9	6505.9	—	​	​	​	​	​	​
10	7336.9	—	​	​	​	​	​	​
11	7353.6	P82462	Muscarinic toxin like protein 1	*N*. *kaouthia*	7357.5	No	Showed practically no affinity for muscle type nAChR	Kukhtina et al. (2000)
12	6689.8	P60305	Cytotoxin 1	*N*. *kaouthia*	6693.1	Yes (as monomer)	Heterodimer Cbtx: Yes *Torpedo* and α7 nAChRs	Osipov et al. (2008)
13	6704.8	P01446	Cytotoxin 3	*N*. *kaouthia*	6709.1	Yes (as monomer)	Heterodimer Cbtx: Yes *Torpedo* and α7 nAChRs	Osipov et al. (2008)
14	6720.0	—	​	​	​	​	​	​
15	6735.0	P60303	Cytotoxin 4	*N*. *kaouthia*	6739.2	Yes, membrane disruption (pore-forming)	No	By similarity
16	6749.2	A0A7T7DMY7	Cytotoxin 1	*N*. *sumatrana*	6755.2	Yes, membrane disruption (pore-forming)	No	Chong et al. (2020)
17	6764.0	—	​	​	​	​	​	​
18	6850.0	P59276	Cobrotoxin-c	*N. kaouthia*	6850.6	No	Yes	Meng et al. (2002)
19	6843.6	P0DSN0	Nakoroxin	*N*. *kaouthia*	6845.7	No	Ambiguous	Osipov et al. (2017)
20	6857.9	—	​	​	​	​	​	​
21	6875.0	—	​	​	​	​	​	​
22	6884.6	—	​	​	​	​	​	​
23	7610.3	P82935	Tryptophan containing weak neurotoxin	*N*. *kaouthia*	7613.9	No	Competitive antagonist muscle-type nAChR and α7 (weak µM range)	Lyukmanova et al. (2016)
24	7625.8	—	​	​	​	​	​	​
25	7816.4	P01391	α-cobratoxin	*N*. *kaouthia*	7820.9	No	Monomer: Muscle (α1β1γδ)-type nAChR and neuronal (α7)	Osipov et al. (2012)
26	6919.2	—	​	​	​	​	​	​
27	6946.7	P60771	Cobrotoxin	*N*. *kaouthia*	6948.5	No	Muscle-type nAChR	By similarity
28	6962.2	—	​	​	​	​	​	​

Cases involving toxins with highly similar molecular masses that could not be unambiguously distinguished based on MALDI-TOF, data alone are discussed in Supplementary Material 3, where assignments are supported by proteomic abundance and chromatographic distribution.

The distributions shown in Figure 4 therefore integrate both the descriptive MALDI-TOF signal partitioning and the molecular identification obtained by shotgun proteomics, and represent the mean relative intensities calculated across all analyzed fractions. Together, these complementary datasets reveal four distinct distribution profiles following affinity capture on nAChR-containing membranes. Several signals (Figure 4A) were exclusively observed in the unbound fraction across all analyzed venom fractions, indicating the absence of detectable capture under the experimental conditions. A second group of toxins (Figure 4B) showed a predominant distribution in the UF despite partial detection in the BF, indicating limited detectable BF recovery under these conditions. In contrast, most identified toxins showed preferential enrichment in the BF (Figure 4C). Cytotoxin 1 (12, P60305), cytotoxin 3 (13, P01446), cytotoxin 4 (15, P60303), the cytotoxin 1 isoform from *Naja sumatrana* (16, A0A7T7DMY7), nakoroxin (19, P0DSN0), and the expected α-cobratoxin (25, P01391) showed BF-predominant MALDI signals, with mean normalized BF signals above 80%. Similarly, signals 14, 17, 20, 21, and 22 also showed marked BF predominance. Cobrotoxin-c (18, P59276), a tryptophan-containing weak neurotoxin (23, P82935), and signal 24 displayed more balanced distributions between UF and BF while remaining predominantly enriched in the BF overall. A final group of toxins was detected in only a single venom fraction but exhibited predominant enrichment in the BF (Figure 4D). This group included cobrotoxin (27, P60771), signals 26 and 28. Among these, signal 28 was detected predominantly in the BF.

**FIGURE 4:**
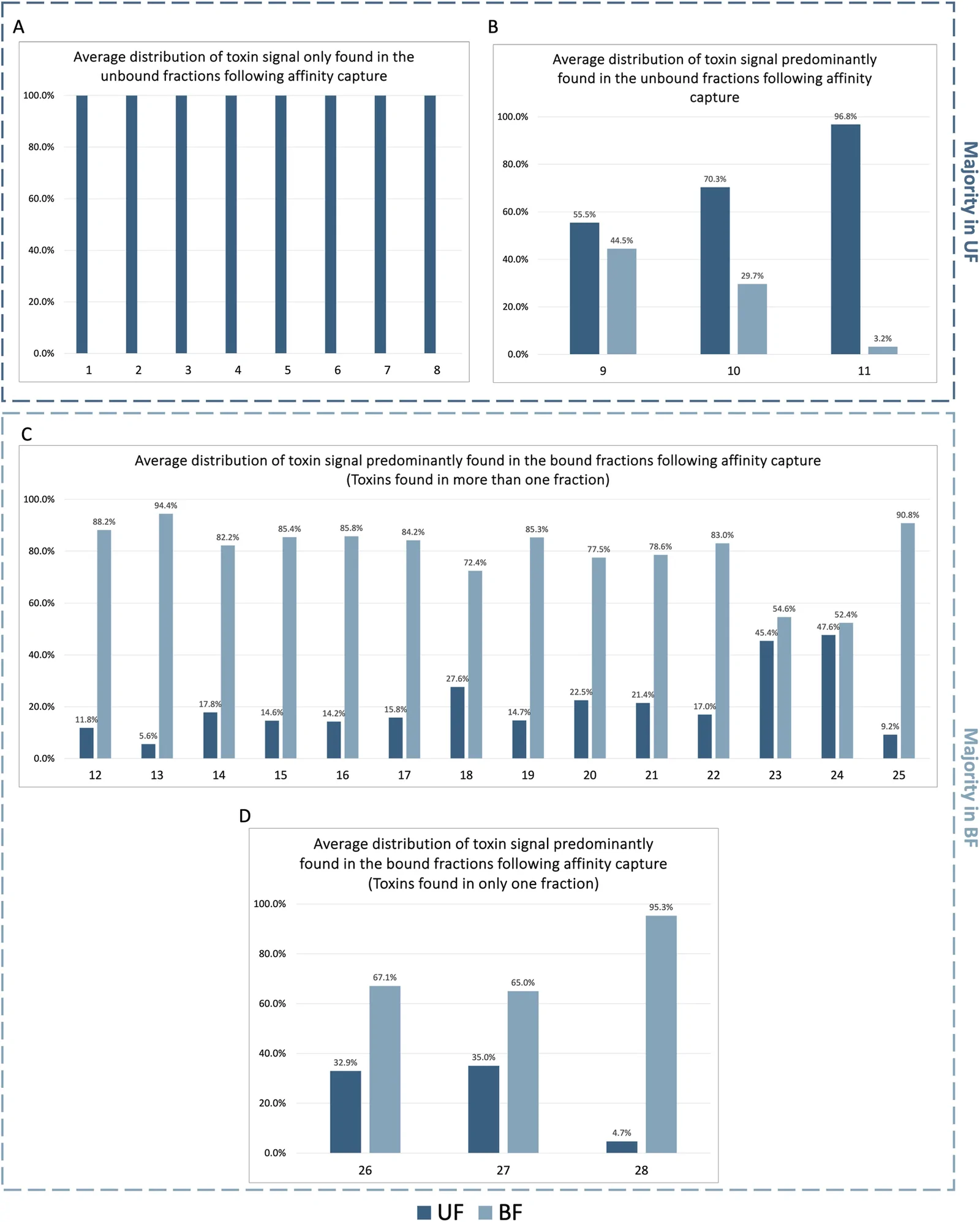
Average distribution patterns of MALDI-TOF toxin signals between unbound and bound fractions following affinity capture of *Naja kaouthia* venom fractions. For each toxin signal within each analyzed chromatographic fraction, UF and BF intensities were normalized to their sum. Bars show the mean normalized signal across the fractions in which that signal was detected, or the value from the single fraction in which it was detected. Each signal is denoted by a number that corresponds to the associated mass and, when available, its protein identification as detailed in Table 2. **(A)** Signals exclusively detected in the UF (100% UF). **(B)** Signals distributed between UF and BF with a predominance in the UF. **(C)** Signals distributed between both fractions but predominantly detected in the BF. **(D)** BF-predominant signals detected in only one chromatographic fraction. Individual results for each chromatographic fraction are shown in Supplementary Figure S7. The plotted MALDI data are from one representative experimental series among the three performed on separate days; means were calculated across chromatographic fractions within that series.

Altogether, the signal distribution patterns revealed a broad diversity of toxin behaviors during affinity capture, ranging from no detectable BF signals to a marked predominance of the BF signal. Several toxins previously reported to interact with muscle-type nAChRs displayed marked BF enrichment, whereas other toxins lacking known nAChR affinity also exhibited substantial retention by the membrane preparation.

### 3.3 *Naja kaouthia* crude venom

The next step was to evaluate whether the affinity-capture method could be applied directly to crude venom, which contains hundreds of distinct molecular components. This could represent a critical improvement, as circumventing the fractionation process offers notable practical advantages. Fractionation and subsequent individual screening of each fraction are both time-consuming and resource-intensive, particularly when handling complex venom mixtures. Direct incubation of crude venom with receptor-rich membranes allowed assessment of toxin recovery and detection in the complete venom mixture. This streamlined approach could significantly accelerate the screening of natural mixtures for bioactive compounds. *N*. *kaouthia* crude venom was then incubated with nAChR membrane preparations. The reference MALDI-TOF spectrum (Figure 5, top) provides a broad overview of the venom composition, displaying several tens of mass signals ranging from m/z 2,000 to 15,000. Comparison of the unbound (Figure 5, middle) and bound (Figure 5, bottom) fractions reveals differences in signal profiles; some are significantly enriched in the bound fraction, while others are either absent or markedly reduced, suggesting a selective capture behavior. A focused view of the relevant mass range highlights peaks corresponding to known neurotoxins.

**FIGURE 5:**
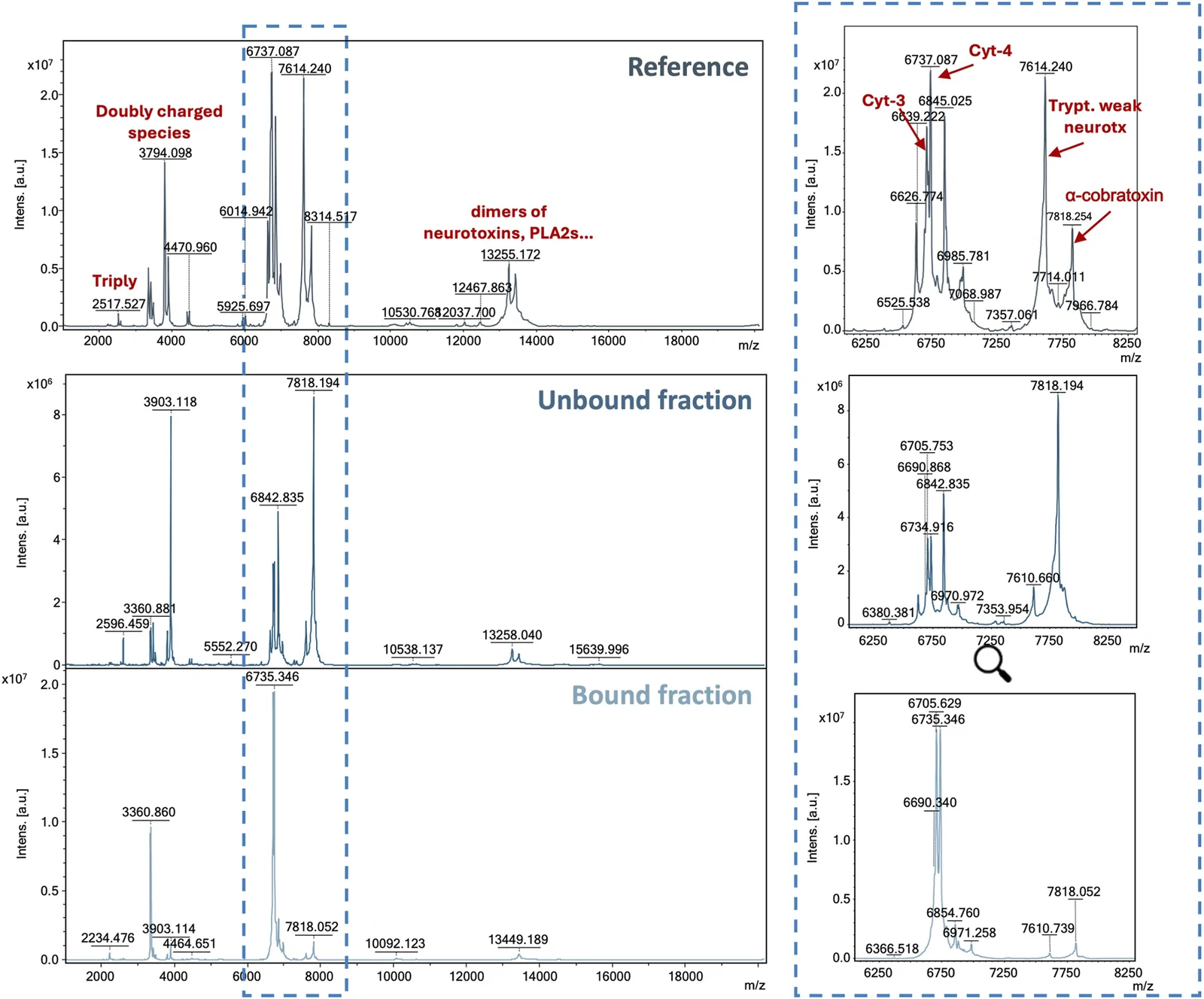
Mass spectrometry comparison of bound and unbound fractions with crude *Naja kaouthia* venom following capture with *Torpedo* electrocyte membranes. MALDI-TOF mass spectra of (i) the reference (no receptor), (ii) the UF, and (iii) the BF, with a zoom on the m/z region of interest. Several toxin-related signals are differentially represented between the two fractions. Specific peaks appear enriched in the BF, while others are predominantly or exclusively present in the UF. No visible peak attributable to membrane denaturation was observed (Supplementary Figure S1C). Spectra from one representative affinity-capture experiment are shown.

To characterize toxin recovery from crude venom, the bound fractions obtained with *Torpedo* and CHO membranes were each compared with crude venom and then compared directly with one another by label-free proteomics (Figure 6). Distinct enrichment profiles were observed depending on the analyzed fraction and membrane preparations used during the capture workflow. Comparison of the *Torpedo*-BF with crude venom identified several enriched toxin groups, including membrane-active cytotoxins (Figure 6A). α-Cobratoxin (P01391) was more abundant in the BF than in crude venom, but did not meet the permutation-based FDR threshold. Cytotoxin 4 (P60303) and cytotoxin 2 (P01445) were enriched relative to crude venom with both *Torpedo* and CHO membranes (Figures 6A,B). The P01446; P24779 protein group, which includes cytotoxin 3 and 5, was enriched relative to crude venom with both membrane preparations. Cytotoxin 1 (P0CH80) was enriched in the CHO-BF relative to crude venom. In the direct comparison, P0CH80 was the majority protein ID of the P0CH80; Q9DGH9 protein group, which was more abundant in the CHO-BF than in the *Torpedo*-BF (Figure 6C). α-Cobratoxin, nakoroxin (P0DSN0), and cytotoxin 1 from *N. sumatrana* (A0A7T7DMY7) were more abundant in the *Torpedo*-BF, whereas the tryptophan-containing weak neurotoxin (P82935), cytotoxin 1 (P60305), and the P01446; P24779 protein group were more abundant in the CHO-BF. Cytotoxin 4 did not show a significant difference between the two bound fractions. The toxin groups discussed in the direct BF comparison had three measured LFQ values per membrane preparation. The P14613; P60771 protein group was quantified in all three *Torpedo*-BF samples and all three crude-venom samples, but in none of the CHO-BF samples. Its statistical classification in Figures 6B,C therefore relies on imputation of the three CHO-BF values. Accordingly, single-accession names were retained only when supported at that level by the proteomic evidence, whereas unresolved three-finger toxin isoforms were reported as protein groups. Definitive isoform-level characterization of these groups would require toxin purification followed by orthogonal sequence-resolving analyses. PCA provided an overview of the proteomic profiles of crude venom and the fractions obtained with each membrane preparation (Supplementary Figure S10). Tables 3 and 4 summarize previously reported toxin activities.

**FIGURE 6:**
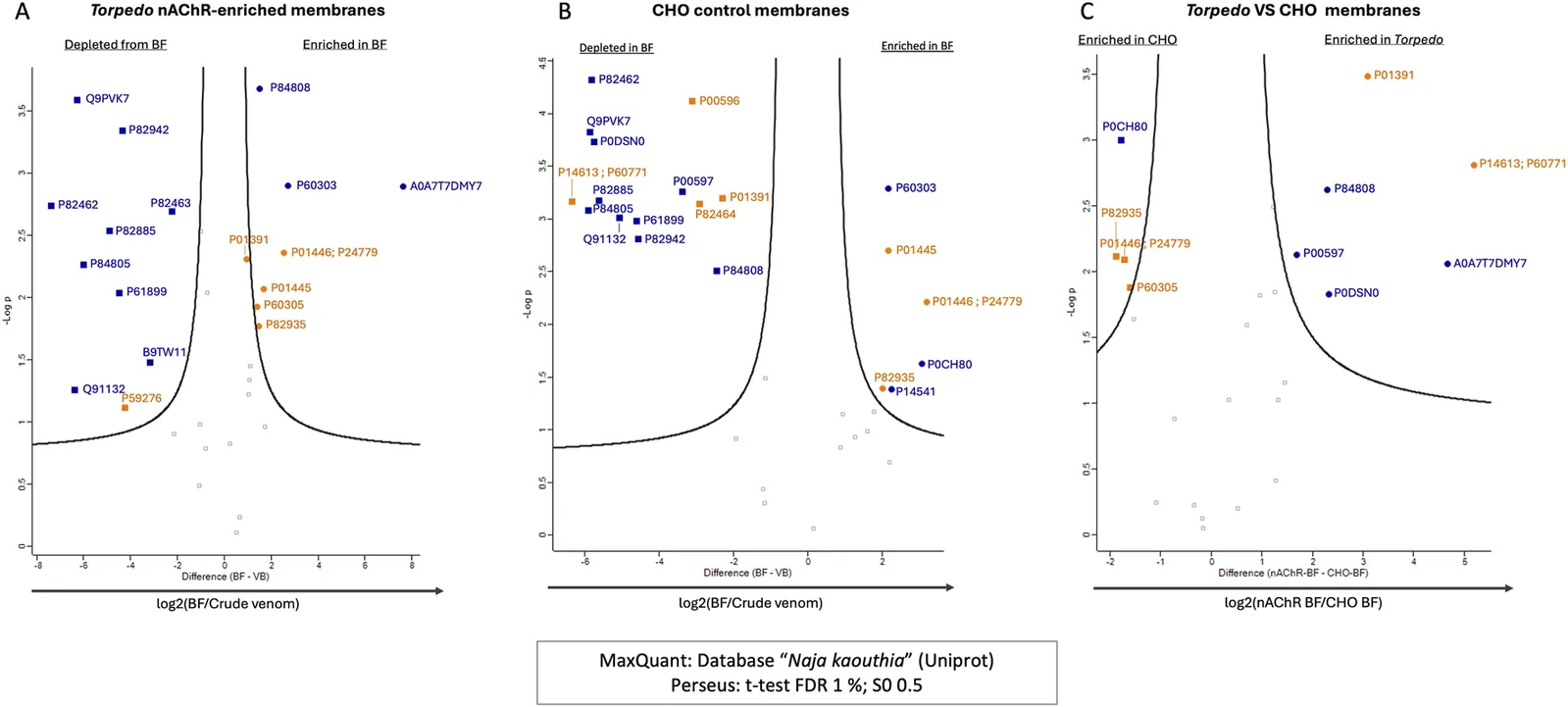
Differential proteomic analysis of crude *Naja kaouthia* venom following affinity capture using *Torpedo* electrocyte membranes or CHO cell membranes. **(A)** *Torpedo*-BF compared with crude venom. **(B)** CHO-BF compared with crude venom. Positive log_2_ differences in panels A and B indicate greater abundance in the bound fraction than in crude venom. **(C)** Direct comparison of *Torpedo*-BF and CHO-BF. Positive log_2_ differences in panel C indicate greater abundance in the *Torpedo*-BF, whereas negative differences indicate greater abundance in the CHO-BF. Orange labels indicate toxins with previously reported nAChR-related activity, and blue labels indicate toxins without such reported activity. These annotations describe prior knowledge and do not establish the mechanism of retention observed in this study. For cytotoxins, reported nAChR-related activity may concern α-cobratoxin-containing heterodimers. Labels correspond to the majority protein IDs reported by MaxQuant for each protein group. The curved thresholds were calculated in Perseus using a permutation-based FDR of 1% and S0 = 0.5.

**TABLE 3:** Proteins and protein groups associated with known or reported nAChR activity identified in the volcano plot analyses.

(Group) accession number (UniProt)	Name	Cytotoxicity	Binding to nAChRs
P00596	Acidic phospholipase A2 CM-II	—	Competitive antagonist muscle-type nAChR and α7 (weak µM range)
P01391	α-cobratoxin	—	Monomer: Muscle (α1β1γδ)-type nAChR and neuronal (α7)
P01445	Cytotoxin 2	Monomer: Yes	Heterodimer Cbtx: *Torpedo* and α7 nAChRs
P01446; P24779	Cytotoxin 3; Cytotoxin 5	Cytotoxin 3: Monomer: Yes Cytotoxin 5: Yes	Cytotoxin 3: Heterodimer Cbtx: *Torpedo* and α7 nAChRs Cytotoxin 5:/
P14613; P60771	Short-neurotoxin 1; cobrotoxin	—	Both: Muscle-type nAChR
P59276	Cobrotoxin-c	—	Yes
P60305	Cytotoxin 1	Monomer: Yes	Heterodimer Cbtx: *Torpedo* and α7 nAChRs
P82464	Muscarinic toxin like protein 3	—	Competitive antagonist muscle-type nAChR and, with highest affinity, α7
P82935	Tryptophan containing weak neurotoxin	—	Competitive antagonist muscle-type nAChR and α7 (weak µM range)

**TABLE 4:** Proteins without established muscle-type nAChR activity identified in the volcano plot analyses.

Accession number (UniProt)	Name	Cytotoxicity
A0A7T7DMY7	Cytotoxin 1 (*N. sumatrana*)	Membrane disruption (pore-forming)
B9TW11	Zinc finger homeobox protein	—
P0CH80	Cytotoxin 1	Dose- and time-dependent cytotoxicity toward leukemia cells
P0DSN0	Nakoroxin	—
P00597	Acidic phospholipase A2 2	—
P14541	Cytotoxin homolog	Low cytotoxic activity
P60303	Cytotoxin 4	Membrane disruption (pore-forming)
P61899	Venom nerve growth factor	—
P82462	Muscarinic toxin-like protein 1	—
P82463	Muscarinic toxin-like protein 2	—
P82885	Thaicobrin	—
P82942	Hemorrhagic metalloproteinase-disintegrin-like kaouthiagin	—
P84805	Cysteine-rich venom protein kaouthin-1	—
P84808	Cysteine-rich venom protein kaouthin-2	—
Q91132	Cobra venom factor	—
Q9PVK7	Zinc metalloproteinase-disintegrin-like cobrin	—

## 4 Discussion

### 4.1 Capture of small to large toxin ligands for G-Protein coupled receptors: from isolated compounds to complex mixtures

#### 4.1.1 Validation of the affinity-capture workflow using AVP and hV2R

The affinity capture methodology monitored by mass spectrometry was first validated on GPCRs using human vasopressin V2 receptors and arginine vasopressin, its endogenous ligand. The successful enrichment in AVP (M = 1084.2 Da) of the bound fraction demonstrated that the workflow efficiently preserves and detects specific receptor-ligand interactions involving peptide ligands. The experiments were performed in the presence of a complex peptide background to challenge the specificity of the capture process. Despite this highly peptide-rich environment, the background remained exclusively detected in the unbound fraction, whereas AVP was selectively retained by hV2R-overexpressing membranes. These observations strongly support the selectivity of the affinity capture strategy and indicate that receptor-mediated ligand enrichment can be achieved even in the presence of abundant non-specific peptide species. The specificity of the interaction was further reinforced by the negative control experiments performed using CHO membranes lacking hV2R overexpression. Under identical incubation and elution conditions, AVP was no longer detected in the BF, demonstrating that its retention requires the presence of the hV2R receptor rather than non-specific membrane association. The only signal consistently observed in BF controls corresponded to a low-intensity background peak also detected in blank conditions, most likely resulting from membrane degradation products released during acidic elution.

#### 4.1.2 Capture of MQ-1 and AVP-dependent changes in toxin recovery

To further evaluate the versatility of the workflow, the methodology was thereafter challenged using mambaquaretin-1, a larger disulfide-rich venom toxin (∼7 kDa), originally isolated from *D. angusticeps* venom and previously described as a highly selective antagonist of hV2R. In contrast to AVP, MQ-1 belongs to the Kunitz-type toxin family and therefore represents a structurally and functionally distinct class of peptide ligands. The successful capture of MQ-1 demonstrates that the affinity-based workflow is not restricted to small (endogenous) peptides but can also accommodate larger, highly structured venom-derived ligands. The experiments were performed using an RP-HPLC fraction containing MQ-1. Under these conditions, a clear enrichment of the ligand was observed in the BF, accompanied by a substantial decrease in its signal in the UF relative to the reference fraction. This enrichment profile clearly indicates preferential capture of MQ-1 by hV2R-containing membranes. The analysis of the MALDI-TOF spectra additionally revealed a weak BF signal assigned to H-toxin TA2 (P01404) by proteomic analysis. Unlike MQ-1, H-toxin TA2 remained predominantly detected in the UF, while only a weaker signal was observed in the BF. This lower BF signal indicates less efficient recovery under the assay conditions but does not establish a lower affinity for hV2R. H-toxin TA2 was not detected in the BF of the CHO control, and its BF signal decreased upon AVP addition. Together, these observations warrant further investigation of a possible hV2R-associated interaction, while leaving open contributions from other components of the receptor-expressing membrane preparation. MQ-1 was likewise absent from the CHO BF, consistent with its established hV2R pharmacology.

Increasing AVP reduced the recovery of MQ-1 in the BF of hV2R-expressing membranes. This observation is consistent with competition between AVP and MQ-1 but cannot distinguish direct overlap of their binding sites from steric interference or negative allosteric coupling. Independently, the cryo-EM structure reported by Fouillen et al. places the high-affinity MQ-1^K39A^ variant within the V2R orthosteric pocket (Fouillen et al., 2025). Thus, the structural study supports an orthosteric binding mode for this MQ-1 variant, whereas the present affinity-capture experiment documents AVP-dependent redistribution of MQ-1 without establishing its binding pose. H-toxin TA2 showed a low BF signal under control conditions that decreased further upon AVP addition. The AVP-dependent decrease in the H-toxin TA2 BF signal warrants further investigation using purified toxin and receptor-specific binding assays to determine whether H-toxin TA2 interacts directly with hV2R and, if so, to characterize its binding mode.

These experiments also illustrate one of the major advantages of combining affinity capture with mass spectrometry. Unlike conventional radioligand or fluorescence-based competition assays, mass spectrometry enables direct, label-free monitoring of multiple ligands simultaneously with high molecular specificity, even within complex venom-derived mixtures. The AVP-dependent changes in MQ-1 and H-toxin TA2 signals illustrate how affinity capture coupled to mass spectrometry can follow the recovery of distinct venom components within the same experimental preparation. Determining receptor occupancy, binding affinity, or the mechanism underlying these changes requires dedicated quantitative binding experiments.

#### 4.1.3 Impact of venom complexity on ligand enrichment efficiency

The comparison between fractionated and crude venom experiments provided additional insight into the operational limits of the methodology. Although MQ-1 was efficiently enriched from the RP-HPLC fraction, it could not be confidently detected in the BF when the workflow was directly applied to crude *D. angusticeps* venom. This difference highlights the difficulty of recovering and detecting a toxin within a complex biological mixture. In crude venom, MQ-1 may be less abundant relative to dominant components, limiting its detection after affinity capture. Its signal may also be affected by ion suppression and by differences in ionization efficiency between crude and fractionated samples. Chromatographic fractionation reduces sample complexity and increases the relative representation of MQ-1 among the compounds analyzed, which can improve its detectability. These observations show the complementary roles of fractionated and crude venom in the workflow. RP-HPLC fractions facilitate the discovery of receptor-associated toxins by reducing analytical interference and improving the detection of less abundant components. Crude-venom experiments then examine whether these toxins can be recovered in the presence of the complete venom mixture. Under those conditions, co-occurring toxins could also influence MQ-1 retention through competition or other interactions, but their contribution cannot be distinguished from differences in abundance and mass spectrometric detection in the present experiments. The absence of a confidently detected MQ-1 signal in the crude-venom BF therefore does not establish an absence of receptor interaction.

### 4.2 Capture of small to large toxin ligands for ligand-gated ion channels: from isolated compounds to complex mixtures

#### 4.2.1 Validation of the affinity-capture workflow using α-conotoxin GI

To challenge the versatility of the affinity-based methodology, the workflow was applied to a completely different receptor–ligand system involving the nicotinic acetylcholine receptor and α-conotoxin GI, a small disulfide-rich peptide with well-documented affinity (IC_50_ = 20 nM) for muscle-type nAChR. Despite its low molecular weight (m = 1438.6 Da) and the presence of abundant background peptides, α-conotoxin GI was efficiently enriched in the bound fraction. These results mirror those previously obtained with AVP and the vasopressin receptor, highlighting the method’s adaptability across both receptor families and ligand sizes.

#### 4.2.2 Toxin retention following affinity capture of fractionated *Naja kaouthia* venom

The workflow was subsequently applied to muscle-type nAChRs to demonstrate the flexibility of the methodology. Affinity capture experiments performed on RP-HPLC fractions from *N*. *kaouthia* venom revealed distinct toxin distribution profiles between bound and unbound fractions, providing valuable insight into both the selectivity and complexity of toxin retention by nAChR-containing membranes. Several toxins previously described as high-affinity muscle-type nAChR ligands were predominantly detected in the BF. Among them, α-cobratoxin (25, P01391), one of the best-characterized antagonists of muscle-type nAChRs, displayed intense BF enrichment, constituting a robust validation of the affinity capture workflow. Interestingly, α-cobratoxin was detected predominantly in fraction 31 (Supplementary Figure S7), where a minor proportion of the signal remained detectable in the UF, which may reflect differences in toxin loading or recovery between fractions. In contrast, signals corresponding most likely to α-cobratoxin isoforms detected in fractions 35 to 38 were recovered exclusively in the BF, with no detectable signal remaining in the UF under these conditions. Similarly, cobrotoxin (27 P60771) was identified exclusively in fraction 25 and was predominantly recovered in the BF (Supplementary Figure S7), further supporting the method’s ability to recover an established neurotoxin from a fractionated venom sample.

The enrichment of tryptophan-containing weak neurotoxin (23, P82935) is also noteworthy. This toxin is described as a weak antagonist of muscle-type and α7 nAChRs in the micromolar range, yet it showed substantial BF enrichment. Its capture may partly result from its relatively high abundance within *N*. *kaouthia* venom (Laustsen et al., 2015). In addition, toxin competition phenomena likely occur within these complex venom fractions. Multiple structurally related toxins coexist in the venom and may compete for overlapping receptor binding sites. Under such conditions, highly abundant weak ligands may still be significantly retained despite lower intrinsic affinity. Nevertheless, this toxin, as well as cobrotoxin-c (18, P59276), remained partially distributed between UF and BF. Differences in toxin abundance across chromatographic fractions, membrane retention, and MALDI signal response may all contribute to these intermediate profiles. Concentration-controlled capture experiments with recovery standards would help determine whether receptor saturation or competition also contributes.

Unexpectedly, several toxins without known affinity for muscle-type nAChRs also exhibited strong BF enrichment. Cytotoxin 4 (15, P60303) and the cytotoxin 1 isoform (16, A0A7T7DMY7) are particularly interesting because both are described as pore-forming toxins capable of membrane disruption through direct interaction with lipid bilayers. Their predominant BF recovery suggests that non-specific membrane association contributes to toxin retention during the affinity capture workflow. This observation indicates that membrane-active toxins may be recovered independently of direct receptor recognition, especially when using native membrane preparations rich in phospholipids and membrane-associated proteins. Similarly, cytotoxin 1 (12, P60305) and cytotoxin 3 (13, P01446), although not considered classical monomeric muscle-type nAChR ligands, have previously been associated with nAChR recognition through heterodimeric complexes involving α-cobratoxin. Association with α-cobratoxin could contribute to the recovery of these cytotoxins from mixed venom fractions, whereas interactions with membrane lipids or other venom components offer additional explanations. The fraction-based signals do not distinguish these possibilities.

Nakoroxin (19, P0DSN0) was predominantly recovered in the BF of the analyzed venom fractions, despite having no established affinity for muscle-type nAChRs. Previous studies demonstrated that nakoroxin does not inhibit α-bungarotoxin binding to muscle or neuronal nAChRs, but unexpectedly enhances α-bungarotoxin binding to the acetylcholine-binding protein (AChBP), a structural homolog of the nAChR extracellular domain (Osipov et al., 2017). These findings raise the possibility of an interaction involving a cholinergic protein without demonstrating binding to muscle-type nAChR in the present assay. Recovery from the fractionated venom could also result from association with membrane components or co-retained toxins. Its behavior in the crude-venom proteomic comparisons is considered below. In contrast, muscarinic toxin-like protein 1 (11, P82462), which reportedly exhibits practically no affinity toward muscle-type nAChRs, was predominantly detected in the UF. This profile agrees with its reported low affinity for muscle-type nAChRs. However, the recovery of membrane-active toxins in other bound fractions shows that BF/UF distribution alone cannot establish receptor-dependent binding.

#### 4.2.3 Proteomic comparison of crude-venom affinity-capture fractions

The three comparisons in Figure 6 help distinguish toxin recovery from the starting venom and differential retention by the two membrane preparations. α-Cobratoxin (P01391) illustrates the value of the direct comparison: its preferential recovery with *Torpedo* membranes agrees with its independently established activity at muscle-type nAChR, even though its increase over crude venom did not meet the FDR threshold in Figure 6A. The recovery of cytotoxin 4 (P60303) and cytotoxin 2 (P01445) with both membrane preparations, together with the CHO-associated enrichment of cytotoxins P0CH80 and P60305 and the P01446; P24779 protein group, indicates that nAChR enrichment is not required to retain these cytotoxins. Given the membrane activity of this toxin family, association with membrane components is a plausible contributor to their recovery. Association with other venom toxins remains possible in the mixed samples. The CHO preference of the tryptophan-containing weak neurotoxin (P82935), despite its reported weak nAChR activity, further shows that known pharmacology does not necessarily predict which membrane preparation retains more toxin under these conditions. Preferential retention by *Torpedo* is therefore most informative when considered alongside the toxin’s known activity and its propensity to interact with membranes. The A0A7T7DMY7 provides an example: its *Torpedo* preference warrants investigation of the membrane properties responsible for its capture, but its membrane-active character provides a plausible explanation independent of direct nAChR recognition. The direct comparison can thus prioritize associations for receptor-specific study while also identifying toxins whose retention is likely influenced by membrane composition.

Nakoroxin (P0DSN0) presents a distinct pattern that complements its recovery in the bound fractions of chromatographic venom fractions discussed above. In the crude-venom analysis, its LFQ signal was significantly lower in the CHO-BF than in the crude-venom sample (Figure 6B), whereas the direct comparison showed significantly greater abundance in the *Torpedo* than in the CHO-BF (Figure 6C). These observations support preferential association with the *Torpedo* preparation without establishing the molecular basis of that association. The reported enhancement of α-bungarotoxin binding to acetylcholine-binding protein by nakoroxin raises the possibility of an interaction involving a cholinergic protein. In another three-finger toxin, WTX, structural and mutational analyses identified contacts with both α7 nAChR and the surrounding lipid membrane (Shenkarev et al., 2022). Receptor and lipid association must therefore both be considered when investigating the retention of nakoroxin. Purified nakoroxin could be tested for concentration-dependent binding to muscle-type nAChR reconstituted in lipid nanodiscs by surface plasmon resonance, using receptor-free nanodiscs of the same lipid composition as a reference. Testing the isolated toxin would exclude retention mediated by other venom components, while comparison with receptor-free nanodiscs would assess its association with lipids. If receptor-associated binding is detected, the effect of nakoroxin on acetylcholine-evoked currents could be measured by electrophysiology. Evidence of binding and functional modulation would support further investigation of its interaction site through structural analysis or mutagenesis.

For other prioritized candidates, parallel capture assays on aliquots of the same *Torpedo* membrane preparation, with and without a site-selective nAChR antagonist, could test whether occupancy of that receptor site changes toxin recovery. Binding experiments with purified toxins and matched receptor-free lipid preparations would help distinguish receptor-associated retention from lipid association.

#### 4.2.4 Concluding remarks on ligand fishing from complex venom mixtures

The comparison between fractionated and crude venom highlights the importance of RP-HPLC separation for toxin discovery. Cobrotoxin (P60771) was identified individually and predominantly recovered in the BF from fraction 25. In the crude-venom proteomic analyses, P60771 was not resolved as an individual protein entry in the volcano plots. Instead, the shared P14613; P60771 protein group was depleted in the CHO BF relative to crude venom (Figure 6B) and more abundant in the *Torpedo* BF than in the CHO BF (Figure 6C). This group did not meet the significance threshold in Figure 6A. Crude-venom analysis thus identified a differential detection pattern for the shared protein group, while fractionation enabled the individual identification and assessment of P60771 in the BF. This need for fractionation is consistent with the analytical constraints of complex mixtures. In MALDI-TOF analysis, abundant components may dominate the spectrum, and matrix-dependent ion suppression can reduce other peptide signals (Perkins et al., 1993). In LC-MS/MS, peptide coelution can cause electrospray ion suppression, while data-dependent precursor selection can limit the identification of less abundant peptides (Budamgunta et al., 2019). These effects may contribute to differences between fractionated and crude samples, although their individual contributions were not measured here. RP-HPLC fractionation is therefore an essential first step for discovering candidate toxins in this workflow. Reliance on crude-venom enrichment plots alone risks overlooking established ligands or leaving closely related toxins unresolved. Subsequent crude-venom analysis remains useful for assessing candidate recovery in the complete mixture and comparing retention by the *Torpedo* and CHO preparations. Any contribution from competition among venom components requires a dedicated experiment.

## 5 Advantages, challenges, and future applications

The application of membrane-based affinity capture to disulfide-rich snake toxins and crude venom, together with quantitative proteomics, supports the prioritization of venom components associated with receptor-containing membrane preparations. Unlike conventional bioactivity-guided fractionation approaches, this target-oriented workflow can screen complex mixtures before extensive purification, while subsequent experiments remain necessary to validate receptor-specific interactions.

One of the main strengths of the workflow is its versatility. The methodology successfully captured ligands interacting with both G protein-coupled receptors and ligand-gated ion channels, while remaining compatible with structurally diverse compounds ranging from small peptides such as AVP to larger disulfide-rich toxins such as MQ-1. The use of membrane preparations preserves receptors within a near-native lipid environment, which may better maintain receptor conformation and physiologically relevant interactions compared with purified recombinant proteins. This aspect is particularly relevant for transmembrane receptors whose activity strongly depends on membrane organization. Another major advantage lies in the integration of label-free mass spectrometry and quantitative proteomics tools. High-resolution MS enables sensitive detection of enriched peptides while simultaneously providing structural information through fragmentation analyses. In parallel, software such as MaxQuant and Perseus facilitates statistical comparison between conditions, allowing rapid prioritization of receptor-enriched toxins within highly complex venom samples.

Despite these advantages, several limitations remain. Cell membrane preparations contain lipid bilayers and endogenous membrane proteins that may contribute to nonspecific interactions, making appropriate controls essential to assess background membrane retention. In addition, highly abundant toxins may dominate MS signals and mask lower-abundance peptides of potential pharmacological interest. Weak or transient interactions may also be lost during washing and centrifugation steps. The methodology detects retained molecules rather than pharmacological activity itself, meaning that downstream functional assays remain necessary to determine whether captured ligands act as agonists, antagonists, or allosteric modulators.

Beyond venom peptide discovery, the broader implications of this methodology are particularly promising. The workflow could potentially be adapted to virtually any membrane receptor for which an overexpression system can be established, thereby extending its applications to endogenous peptide discovery, antibody screening, synthetic peptide libraries, or natural product research. In oncology, such approaches may facilitate the identification of ligands targeting receptors overexpressed in cancer cells, opening perspectives for targeted therapeutics or molecular imaging agents (Chow and King, 2024; Freuville et al., 2026).

Future developments will likely benefit from advances in artificial intelligence and structural bioinformatics. AI-based protein structure prediction and molecular docking tools could help predict receptor–ligand interactions, prioritize candidate toxins, and identify structure–activity relationships before experimental validation (Michino et al., 2025). In parallel, machine learning approaches applied to large proteomic datasets may improve the discrimination of true ligands from nonspecific binders and accelerate venom peptide discovery. The integration of affinity capture methodologies with AI-driven analysis, therefore, represents a promising perspective for the future of receptor-oriented toxin screening.

## 6 Conclusion

Overall, this study demonstrates the feasibility and versatility of an affinity-based mass spectrometry workflow for the recovery and identification of venom toxins retained by receptor-containing membrane preparations. By combining membrane-based affinity enrichment with MALDI-TOF mass spectrometry and proteomic analyses, the methodology enabled the detection of receptor-associated ligands ranging from isolated peptides to crude venom-derived mixtures within a relatively simple and rapid experimental workflow.

The successful enrichment of arginine vasopressin and mambaquaretin-1 on hV2R-overexpressing membranes validated the approach’s capacity to capture ligands with distinct molecular weights and structural properties for a GPCR target. Its application to the *Torpedo* nicotinic acetylcholine receptor further demonstrated the workflow’s adaptability to a structurally different receptor system. Membrane blanks helped identify signals originating from the experimental workflow, while the comparison with CHO membranes revealed toxin retention by a distinct preparation without nAChR enrichment. Because CHO and *Torpedo* membranes do not provide matched backgrounds, these controls support candidate prioritization but do not establish nAChR-dependent binding for newly observed toxin associations.

## Data Availability

The datasets presented in this study can be found in online repositories. The names of the repository/repositories and accession number(s) can be found in the article/Supplementary Material.
