# Supplementary material for: Mass spectrometry-based affinity capture of peptide ligands from complex venom mixtures
Source: Front Chem. 2026 Oct 27;14:1971257. doi: 10.3389/fchem.2026.1971257 (PMC13748256; doi:10.3389/fchem.2026.1971257)
Supplement: Supplementary file 1 [file DataSheet1.pdf]

Supplementary Informations

Mass spectrometry-based affinity capture of peptide ligands from complex venom mixtures

Lou Freuville[1], Rudy Fourmy[2], Aude Violette[2], Alain Brans[3], Romulo Aráoz[4], Nicolas Gilles[4], Loïc Quinton[1*]

[1]Mass Spectrometry Laboratory, MolSys Research Unit, Department of Chemistry, University of Liège, Allée du Six Août, 11 – Quartier Agora, 4000, Liège, Belgium

[2]Alphabiotoxine Laboratory sprl, Montroeul-au-Bois,7911, Belgium

[3]Centre for Protein Engineering, University of Liège, B-4000 Liège, Belgium

[4]CEA, Paris-Saclay University, 91191 Gif-sur-Yvette, France

Corresponding author
Loic.Quinton@

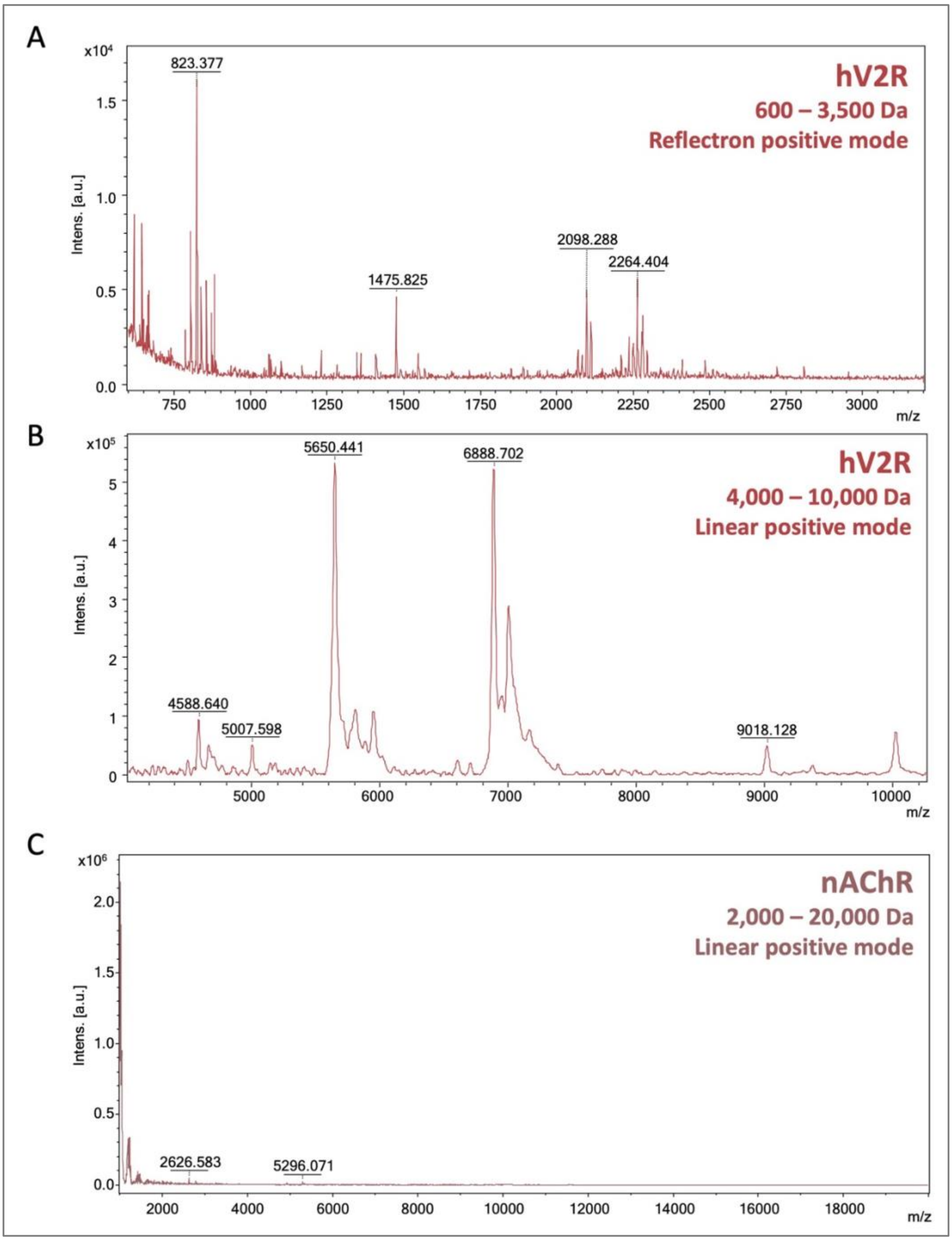

Supplementary Figure 1. MALDI-TOF MS spectra of blank control used for affinity capture. (A) Blank control spectra obtained after complete affinity-capture workflow using membranes overexpressing hV2R, recorded in positive reflectron mode (m/z 600–3,500). **(B)** In positive linear mode (m/z 4,000–10,000). **(C)** Blank control spectrum obtained from nAChR-overexpressing membranes, recorded in positive linear mode (m/z 2,000–20,000). Spectra correspond to eluates recovered after acid treatment (5% TFA) in the absence of added peptides, highlighting signals arising from membrane-associated components released upon denaturation.

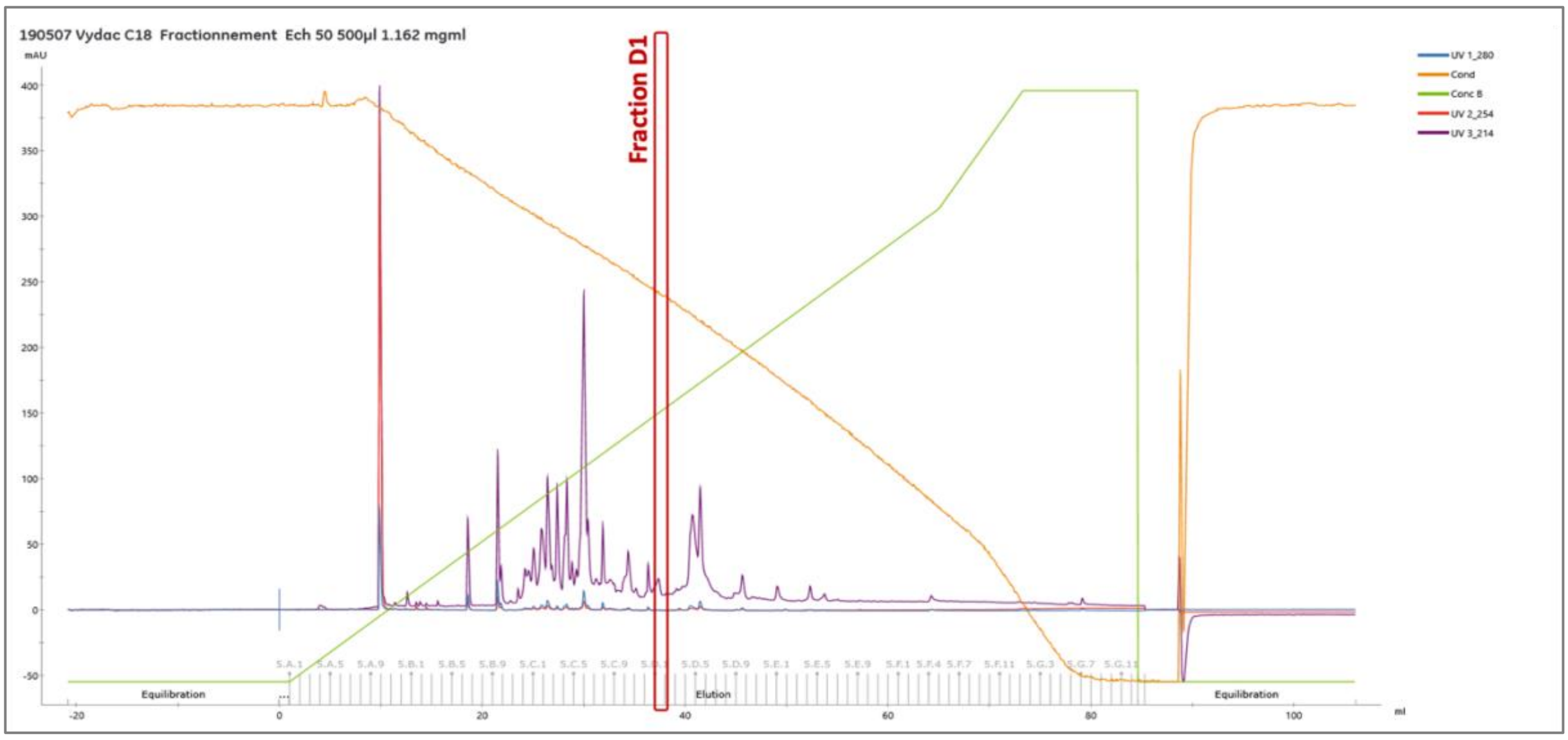

Supplementary Figure 2. RP-HPLC chromatographic profile of *Dendroaspis angusticeps* crude venom and selection of the fraction used for affinity-fishing experiments. The highlighted peak corresponds to the collected fraction used in affinity-capture assays. This fraction contains mambaquaretin-1, the peptide of interest investigated in the present study.

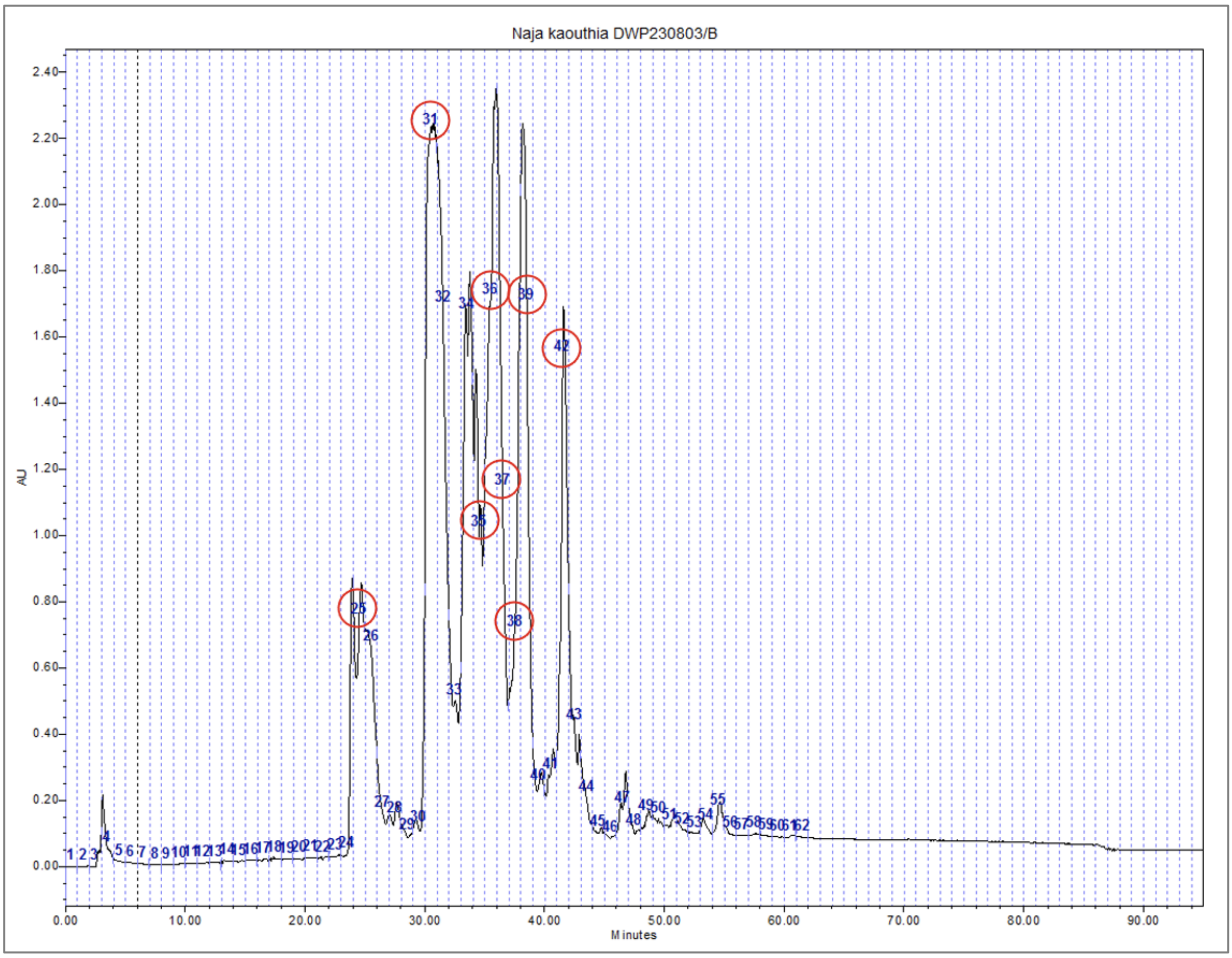

Supplementary Figure 3. RP-HPLC chromatographic profile of *Naja kaouthia* venom with fraction collection strategy. Reversed-phase high-performance liquid chromatography

(RP-HPLC) separation of crude *Naja kaouthia* venom showing the global elution profile. Fractions corresponding to the most intense chromatographic peaks (F25, F31, and F42) were collected based on UV absorbance signal intensity. In addition, consecutive fractions from F35 to F39, encompassing the elution window of α-cobratoxin, were systematically collected due to the known presence of this toxin in this retention time range.

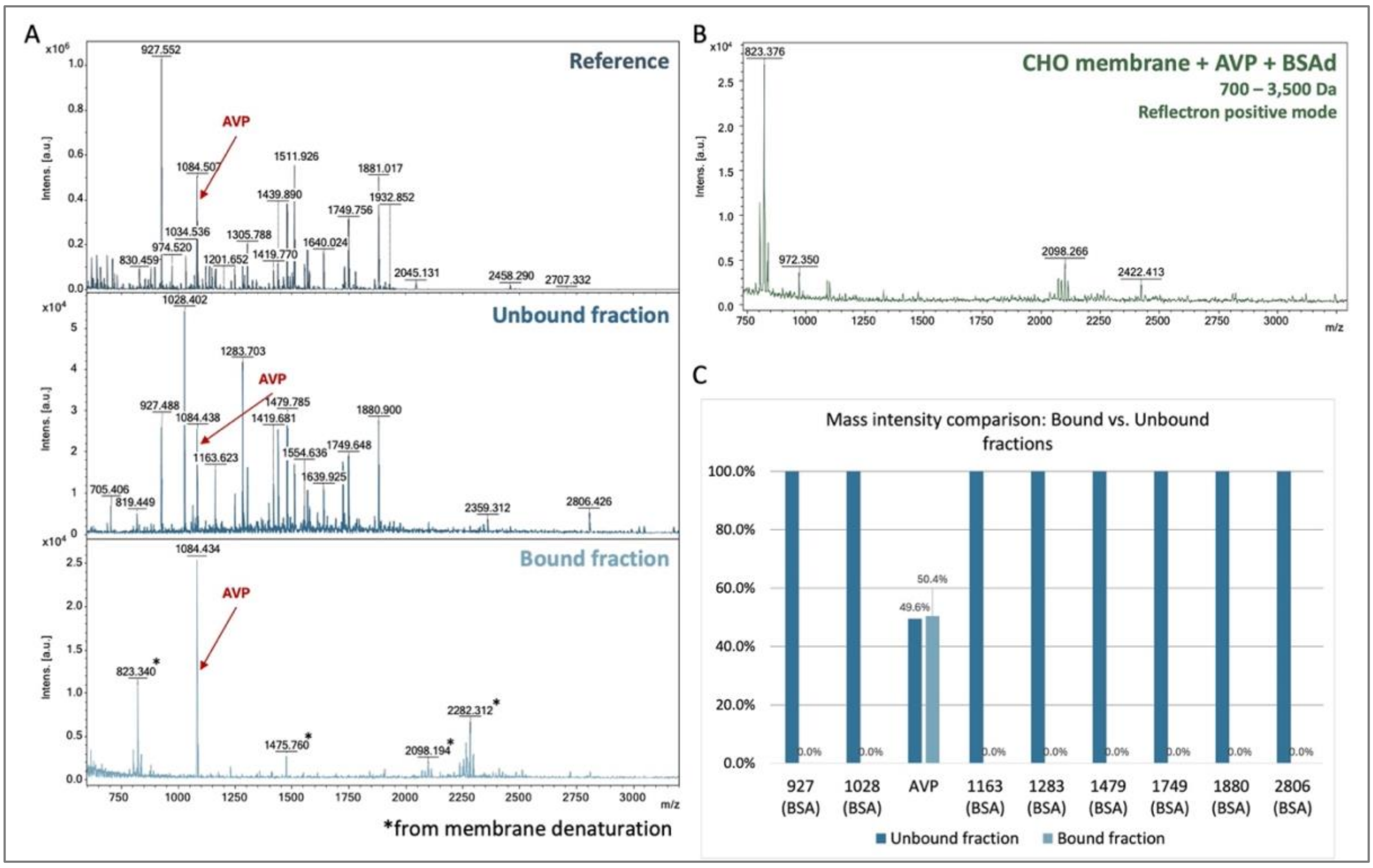

Supplementary Figure 4. Affinity capture of vasopressin on hV2R in the presence of a complex peptide background. (A) MALDI-TOF mass spectra of (i) the reference mixture (AVP spiked in BSA-digested peptides, no receptor), (ii) the unbound fraction, and (iii) the bound fraction. In the BF, only the peak corresponding to AVP is detected, with no signal from BSA-digested peptides. Asterisks label additional peaks corresponding to background molecules released by acid shock elution. Peaks marked with an asterisk (*) correspond to signals originating from membrane denaturation, as determined from the blank control performed using hV2R-expressing membranes in the absence of added peptide ligands (**Supplementary Figure 1A**). **(B)** MALDI-TOF mass spectra of the negative control performed with CHO membranes not expressing the receptor. **(C)** Histogram graph comparing the relative signal intensities of AVP and BSA peptides in the UF (dark blue) and BF (light blue). Bars represent normalized intensity values, confirming selective capture of AVP by hV2R and absence of BSA peptides.

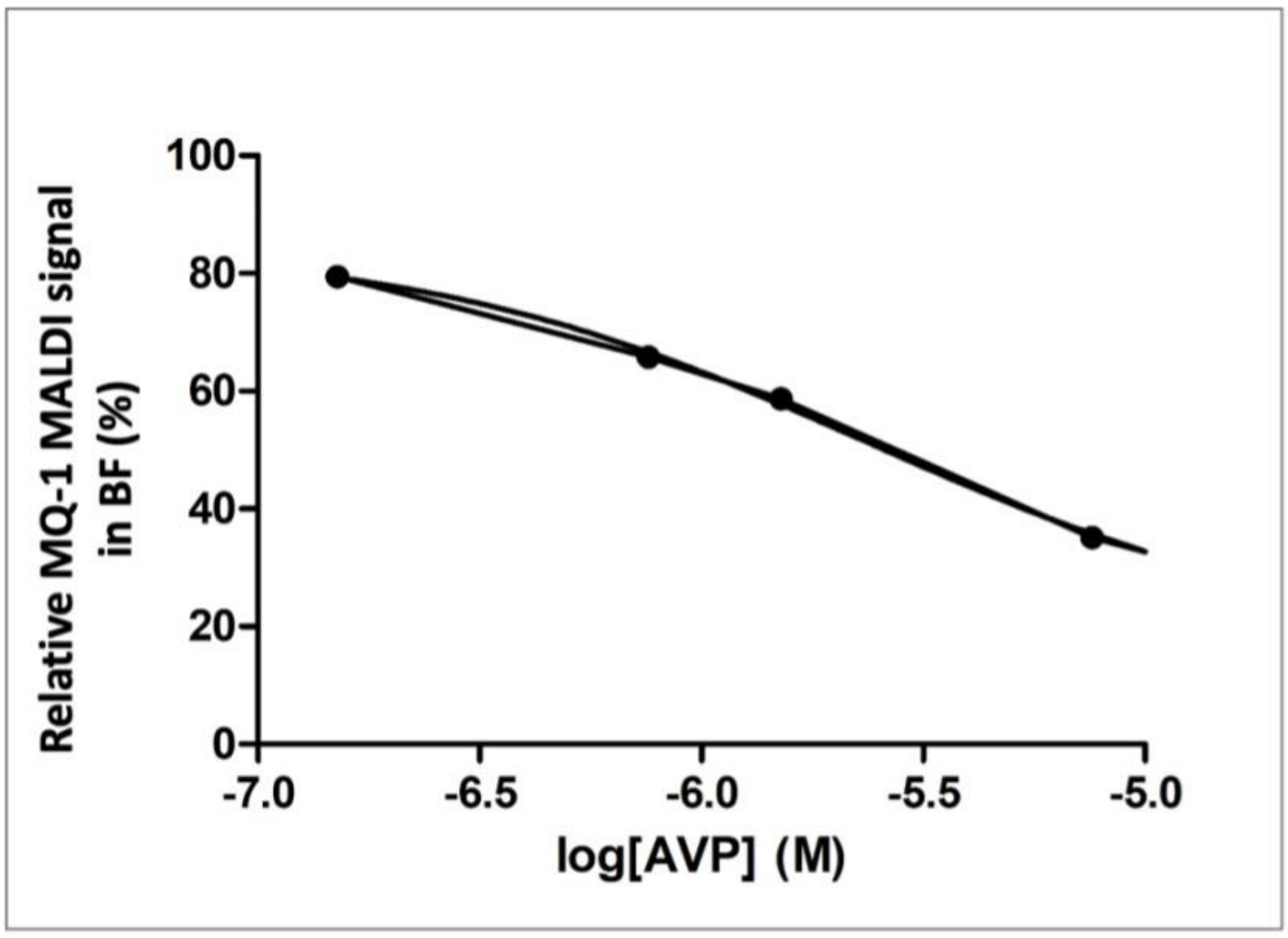

Supplementary Figure 5. AVP-dependent change in MQ-1 recovery during hV2R affinity capture. Nonlinear regression of the normalized MQ-1 BF MALDI signal as a function of AVP concentration. The curve is provided for qualitative illustration only and does not represent a quantitative determination of binding affinity.

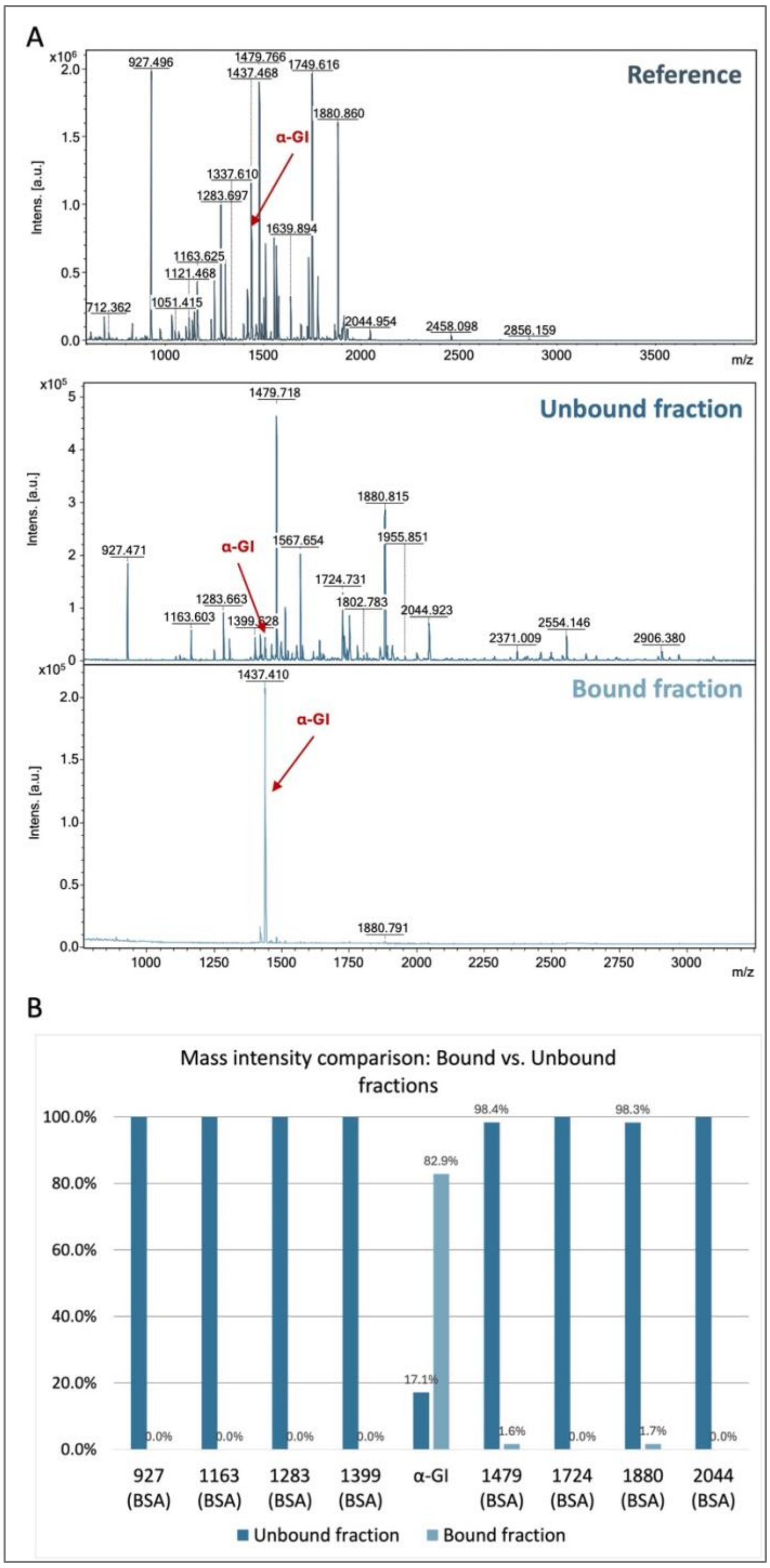

Supplementary Figure 6. Affinity capture of α-conotoxin GI on nAChR in the presence of a complex peptide background. (A) MALDI-TOF mass spectra of (i) the reference mixture (α-conotoxin GI and BSA-digested peptides, no receptor), (ii) the UF, and (iii) the BF. The α-conotoxin GI signal is reduced in the UF and enriched in the BF. Most BSA-derived peptides remain in the UF; only one is slightly detected in the BF. **(B)** Histogram comparing the relative signal intensities of α-conotoxin GI and BSA peptides in the UF (dark blue) and BF (light blue). Bars represent normalized intensity values, confirming selective binding of the toxin to nAChR.

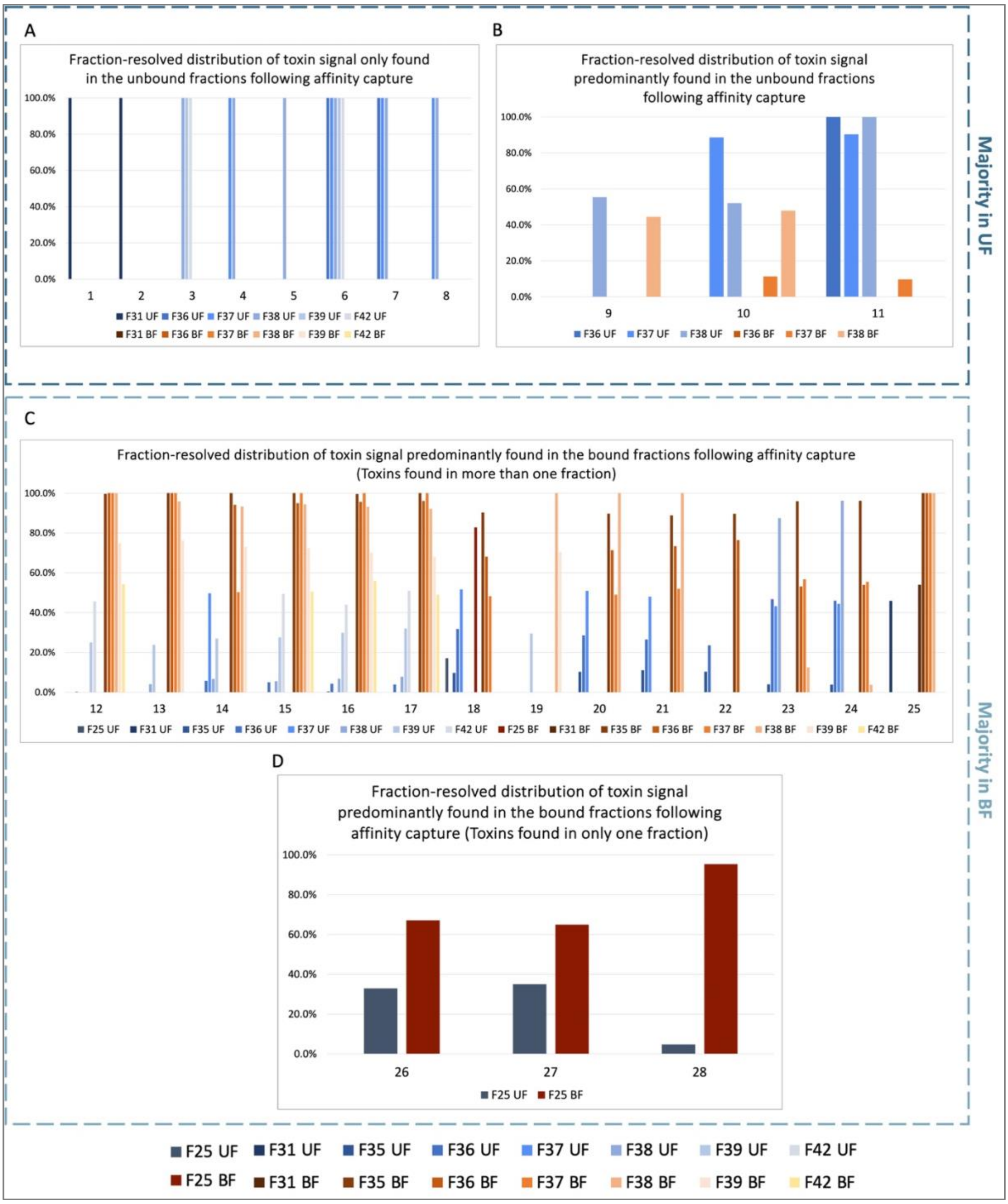

Supplementary Figure 7. Fraction-resolved distribution patterns of toxin signals between unbound and bound fractions following affinity capture of *Naja kaouthia* venom fractions. Bar plots represent the relative signal intensities (%) of individual toxin signals detected in each analyzed venom fraction following affinity capture, calculated from MALDI-TOF MS data and normalized to the total signal detected in both fractions (UF + BF) for a given toxin signal within each venom fraction. Each signal is denoted by a number corresponding to the associated mass and, when available, its protein identification as detailed in **Table 2**. The color code identifies the venom fraction in which the toxin was detected. **(A)** Toxin signals exclusively detected in the UF across all analyzed venom fractions. **(B)** Toxin signals distributed between UF and BF but predominantly detected in the unbound fraction. **(C)** Toxin signals distributed between UF and BF with a predominance in the bound fraction and detected in more than one

venom fraction. **(D)** Toxin signals predominantly detected in the bound fraction and observed in only a single venom fraction. Unlike **Figure 4**, which presents the average distribution patterns calculated across all venom fractions for each toxin signal, this figure displays the individual fraction-specific distributions, highlighting the variability of toxin partitioning among the different venom fractions analyzed.

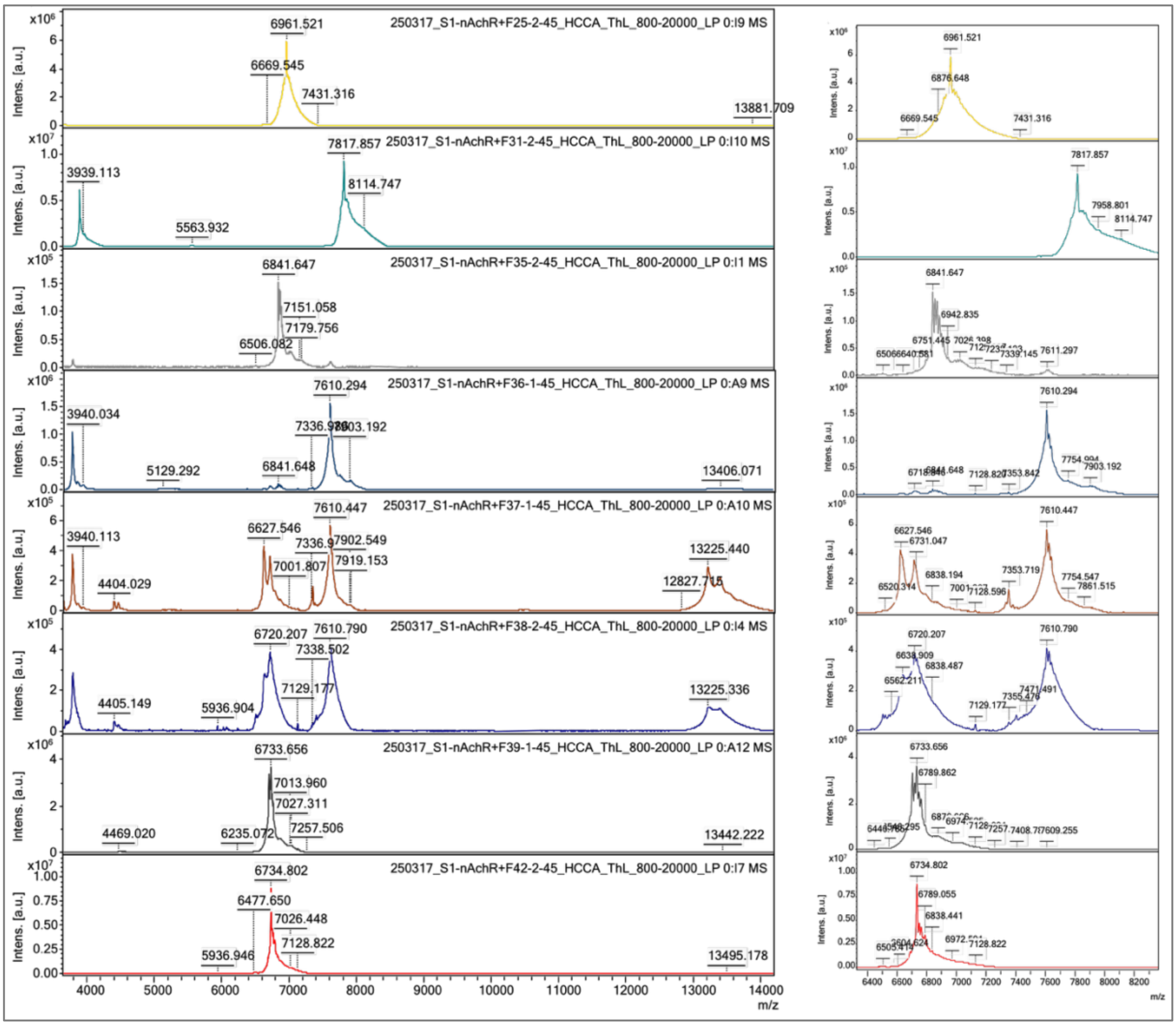

Supplementary Figure 8. MALDI-TOF MS profiles of bound fractions obtained following affinity capture of *Naja kaouthia* venom fractions on nAChR-expressing membrane preparations. Spectra correspond to venom fractions F25, F31, F35, F36, F37, F38, and F42 and are displayed as stacked traces for comparison. The left panel shows the complete mass range analyzed, whereas the right panel presents a magnified view of the 6–8 kDa region to facilitate visualization of low-intensity signals and closely spaced toxin masses. These data provide the experimental MALDI-TOF MS profiles underlying the toxin distributions summarized in **Figure 6** and **Supplementary Figure 7**.

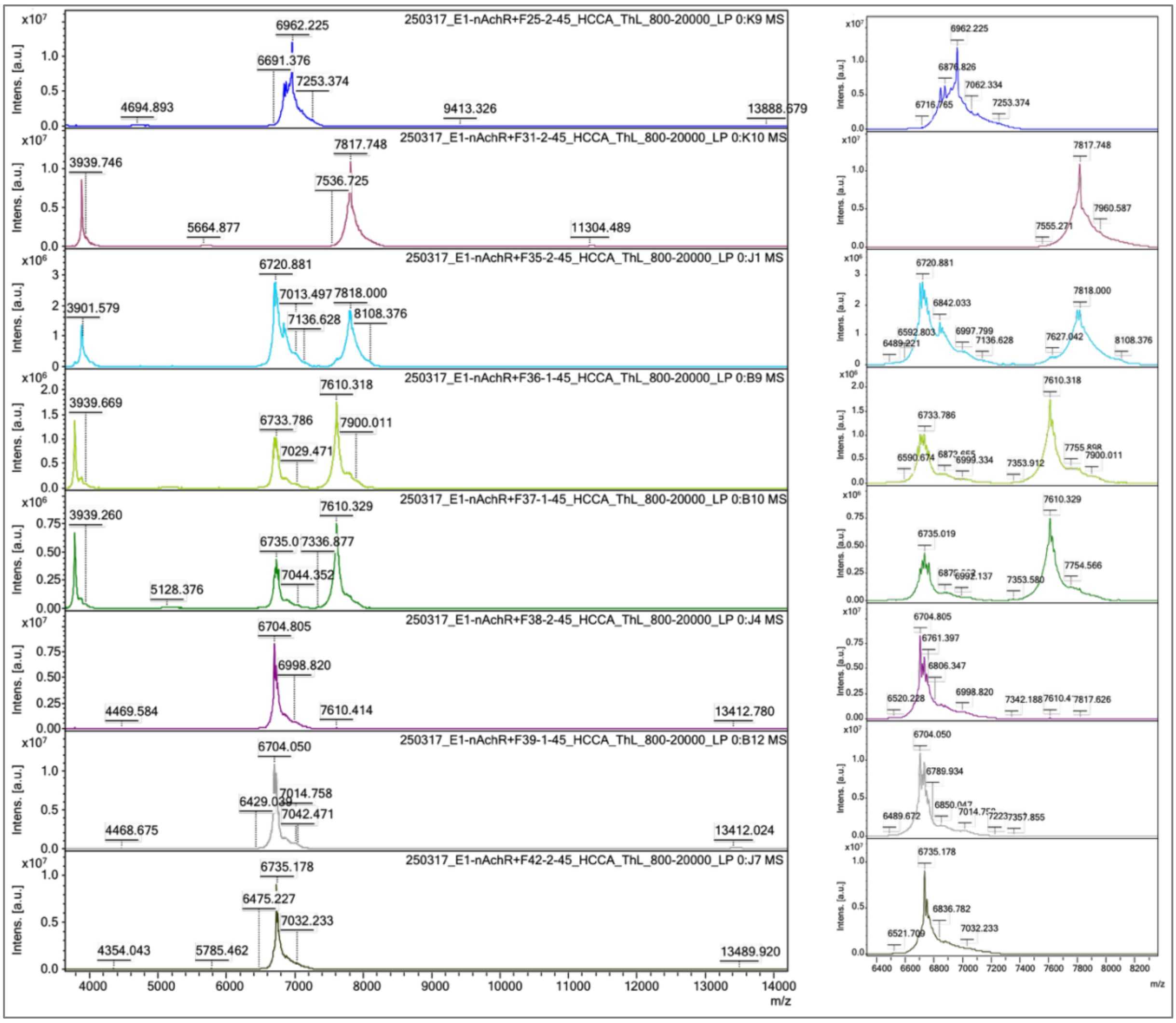

Supplementary Figure 9. MALDI-TOF MS profiles of unbound fractions obtained following affinity capture of *Naja kaouthia* venom fractions on nAChR-expressing membrane preparations. Spectra correspond to venom fractions F25, F31, F35, F36, F37, F38, and F42 and are displayed as stacked traces for comparison. The left panel shows the complete mass range analyzed, whereas the right panel presents a magnified view of the 6–8 kDa region to facilitate visualization of low-intensity signals and closely spaced toxin masses. These data provide the experimental MALDI-TOF MS profiles underlying the toxin distributions summarized in **Figure 6** and **Supplementary Figure 7**.

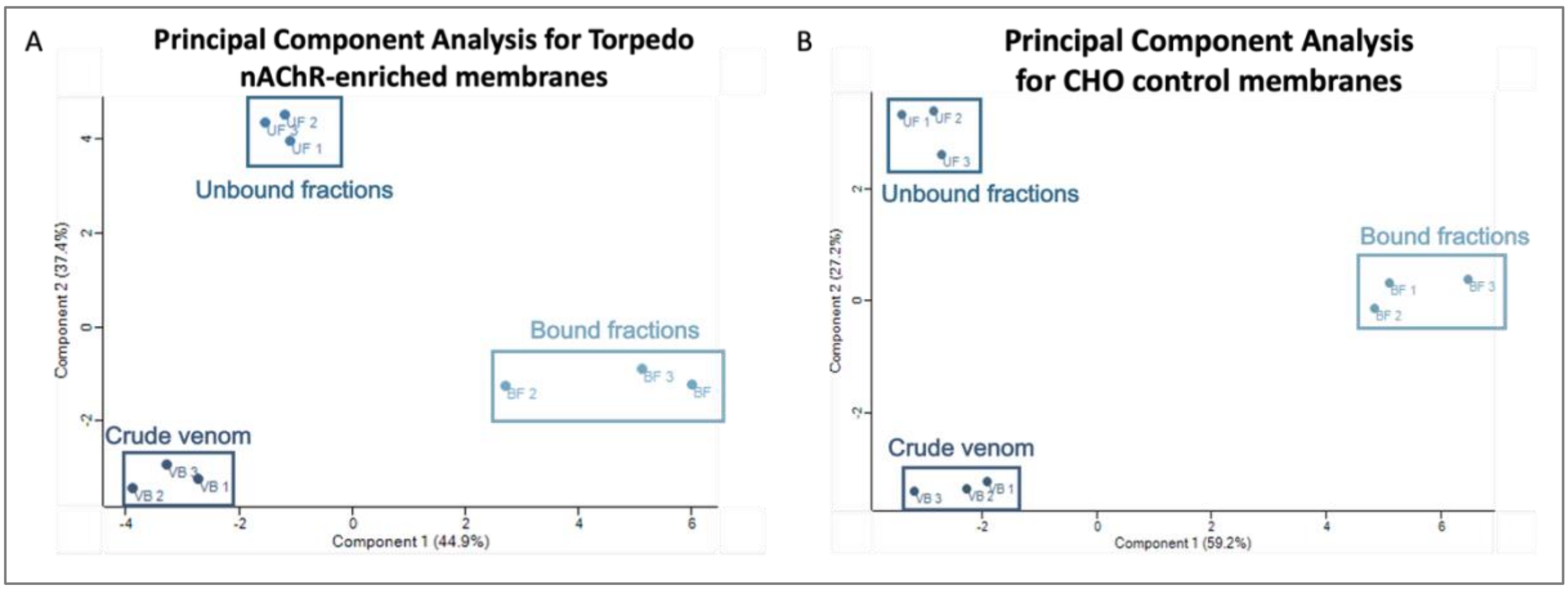

Supplementary Figure 10. PCA of proteomic profiles obtained from affinity capture experiments performed on nAChR-expressing and control CHO membrane preparations. PCA score plots generated from LFQ proteomic data obtained following affinity capture experiments using *N. kaouthia* venom. Protein intensities were processed using MaxQuant and statistically analyzed in Perseus following $\log_2$ transformation and normalization. Each BF and UF point represents one of three experimental affinity-capture replicates, with BF and UF paired within each capture. Each VB point represents one of three technical sample-preparation replicates obtained by separately digesting aliquots of the same crude-venom stock. Crude venom fractions, UF, and BF are shown separately and enclosed in shaded regions for their respective groups. **(A)** PCA of samples obtained from affinity capture experiments performed using *Torpedo* nAChR-enriched membranes. Principal components 1 and 2 explain 44% and 36.5% of the total variance, respectively. The three sample groups (crude venom, UF, and BF) form distinct clusters, indicating substantial differences in protein composition following affinity capture. **(B)** PCA of samples obtained from affinity capture experiments performed using control CHO membrane preparations lacking nAChR overexpression. Principal components 1 and 2 explain 56.5% and 27.3% of the total variance, respectively. Samples remain separated according to their experimental group, although the clustering pattern differs from that observed for nAChR-expressing membranes.

Supplementary Information 1. Detection and identification of toxin ligands for hV2R – Control experiments.

A positive control experiment, inspired by previous work in the lab (Cologna et al., 2015), was designed using vasopressin, the natural ligand of the hV2R. It aims to evaluate the ability of hV2R membrane preparations to selectively capture AVP from a complex peptide background (BSA-digested peptides). Following the affinity incubation, the unbound and bound fractions were collected and analyzed using MALDI-TOF mass spectrometry in reflectron positive mode (**Supplementary Figure 4**). The reference spectrum of AVP spiked into the BSA digest mixture is shown at the top of **Supplementary Figure 4** and illustrates the relative intensity of the AVP signal compared with the other peptide signals. In the unbound fraction (**Supplementary Figure 4A**, middle), AVP remains detectable but shows a reduced signal intensity compared to the reference. This can be interpreted as the result of an excess of vasopressin present in the incubation medium, which remains free in solution and is therefore detected in the unbound fraction. In contrast, no BSA-digested peptides are detected in the bound fraction (**Supplementary Figure 4A**, bottom), while the AVP signal is enriched. A few additional peaks appeared in the bound fraction, corresponding to membrane components released during acid shock elution, as confirmed by control (blank) experiments conducted in the absence of peptide ligands (**Supplementary Figure 4A**).

To assess the specificity of the ligand-receptor interaction, a negative control was performed using CHO cell membranes that do not overexpress the hV2R. The experiment was carried out under the same conditions as the test with receptor-expressing membranes, including the addition of vasopressin and BSA-digested peptides. The resulting MALDI-TOF MS spectrum revealed that no peptide signals were detected in the bound fraction (**Supplementary Figure 4B**). This observation indicates that no non-specific binding to the CHO membrane background is detected in the absence of receptor expression. These results confirm that hV2R-expressing membrane preparations selectively capture their endogenous ligand, AVP, from a complex peptide mixture, demonstrating the specificity and functionality of the system.

Supplementary Figure 4C illustrates the relative distribution of signal intensities for AVP and BSA peptides across the unbound and bound fractions. The data confirm the specific

enrichment of vasopressin in the bound fraction, along with the absence of BSA-digested peptides, further supporting the selective binding capability of the hV2R membrane preparations.

Supplementary Information 2. Detection and identification of toxin ligands for nAChR – Control experiments.

As a positive control experiment, α-conotoxin GI, a well-characterized peptide toxin derived from the venom of *Conus geographus*, was selected. This 13-amino-acid conotoxin specifically targets muscle-type nicotinic acetylcholine receptors, particularly the $(\alpha 1)_2\beta 1\gamma\delta$ subtype found at neuromuscular junctions (see **Table 1**). This positive control assay was designed to confirm the capacity of such nAChR-rich membrane preparations to selectively bind a well-described ligand in a simulated complex molecular background. α-GI was incubated with nAChRs in the presence of BSA-digested peptides, used to mimic a non-specific molecular background. Following incubation, the bound and unbound fractions were analyzed by MALDI-TOF mass spectrometry in reflectron positive mode. The reference spectrum of α-GI spiked into the BSA digest mixture is shown at the top of **Supplementary Figure 6A**. α-GI was detected in the bound fraction (**Supplementary Figure 6A**, bottom), indicating a strong and specific interaction with the nAChR-enriched membranes. While the toxin was also present in the unbound fraction (**Supplementary Figure 6A**, middle), its signal intensity is lower, consistent with high-affinity binding to the receptors. In contrast, most of the BSA-derived peptides remained in the unbound fraction, highlighting the assay selectivity. **Supplementary Figure 6B** illustrates, in a more visual way, the relative distribution of signal intensities for α-GI and BSA peptides across the unbound and bound fractions. These results confirm the selective binding of α-GI under the assay conditions, thereby validating the structural and functional integrity of the receptor preparations used in subsequent experiments and the assay setup as well.

Supplementary Information 3. Assessment of ambiguous toxin assignments based on proteomic abundance and chromatographic distribution.

Three pairs of closely related toxins had theoretical average masses separated by approximately 2 Da and could not be distinguished from the MALDI-TOF intact-mass measurements alone. Peptide-level identifications, chromatographic distributions, and quantitative intensity data from PEAKS were therefore considered together to identify the most likely predominant contributor to each MALDI signal. These assignments do not exclude minor contributions from closely related toxins. Definitive isoform-level characterization would require toxin purification followed by orthogonal sequence-resolving analyses.

The first case involved cytotoxin 2 (P01445, Average theoretical mass = 6737.1 Da) and cytotoxin 4 (P60303, Average theoretical mass = 6739.2 Da). Although these toxins could not be differentiated solely from MALDI-derived masses, their proteomic distributions already suggested distinct behaviors. Cytotoxin 4 was consistently identified across fractions F35–F42, whereas cytotoxin 2 was detected exclusively in fraction F39. This restricted occurrence contrasts with the broad and reproducible presence of cytotoxin 4 throughout the active fraction range. Quantitative PEAKS analysis further reinforced this interpretation, with cytotoxin 4 displaying a markedly higher cumulative peptide intensity ($\approx 1.3 \times 10^8$) than cytotoxin 2 ($\approx 3.28 \times 10^6$). Taken together, the broader chromatographic distribution and substantially higher abundance strongly support cytotoxin 4 as the dominant molecular species associated with the detected signal.

A similar situation was observed for muscarinic toxin-like protein 3 (P82464, Average theoretical mass = 7615.6 Da) and the tryptophan-containing weak neurotoxin (P82935, Average theoretical mass = 7613.9 Da). However, the muscarinic toxin-like protein 3 was detected only in fractions F35–F38, whereas the tryptophan-containing weak neurotoxin was consistently identified across fractions F35–F42, indicating a broader and more stable presence throughout the active chromatographic window. This trend was further supported by PEAKS quantitative analysis, in which the tryptophan-containing weak neurotoxin consistently showed higher peptide intensities across the shared fractions, reaching values up to 4.36×10^8. In contrast, muscarinic toxin-like protein 3 remained substantially less abundant and declined rapidly in later fractions.

This ambiguity was also observed for cobrotoxin-c (P59276, Average theoretical mass = 6850.6 Da) and cobrotoxin II (P82849, Average theoretical mass = 6853.4 Da), whose molecular masses fall within the mass accuracy range of the MALDI-TOF measurements and therefore could not be unambiguously distinguished based on mass data alone. However, their proteomic distributions revealed marked differences in both abundance and chromatographic occurrence. Cobrotoxin-c was consistently identified in fractions F25, F35, F36, and F37, with peptide intensities of 4.7×10^7, 1.5×10^5, 1.85×10^5, and 5.25×10^4, respectively. In contrast, cobrotoxin II was detected exclusively in fraction F25 and at a substantially lower peptide intensity (3.22×10^6). Together, these data favor cobrotoxin-c as the main contributor to the MALDI signal, while a contribution from cobrotoxin II cannot be excluded.
